# Supplementary material for: ‘Sometimes you are forced to play God…’: a qualitative study of healthcare worker experiences of using continuous positive airway pressure in newborn care in Kenya
Source: BMJ Open. 2020 Aug 13;10(8):e034668. doi: 10.1136/bmjopen-2019-034668 (PMC7430418; doi:10.1136/bmjopen-2019-034668)
Supplement: Author's manuscript [file bmjopen-2019-034668.draft_revisions.pdf]

BMJ Open is committed to open peer review. As part of this commitment we make the peer review history of every article we publish publicly available.

When an article is published we post the peer reviewers' comments and the authors' responses online. We also post the versions of the paper that were used during peer review. These are the versions that the peer review comments apply to.

The versions of the paper that follow are the versions that were submitted during the peer review process. They are not the versions of record or the final published versions. They should not be cited or distributed as the published version of this manuscript.

BMJ Open is an open access journal and the full, final, typeset and author-corrected version of record of the manuscript is available on our site with no access controls, subscription charges or pay-per-view fees (http://bmjopen.bmj.com).

If you have any questions on BMJ Open's open peer review process please email info.bmjopen@bmj.com

BMJ Open

"Sometimes you are forced to play God…": a qualitative study of healthcare worker experiences of using continuous positive airway pressure in newborn care in Kenya.

Journal:	*BMJ Open*
Manuscript ID	bmjopen-2019-034668
Article Type:	Original research
Date Submitted by the Author:	02-Oct-2019
Complete List of Authors:	Nabwera, Helen; Liverpool School of Tropical Medicine, International Public Health<br>Wright, Jemma; Betsi Cadwaladr CHC, Paediatric Department<br>Patil, Manasi; Liverpool School of Tropical Medicine<br>Dickinson, Fiona; Liverpool School of Tropical Medicine<br>Godia, Pamela; Liverpool School of Tropical Medicine<br>Maua, Judith; Liverpool School of Tropical Medicine<br>Sammy, Mercy; Gertrude's Garden Children's Hospital<br>Naimoi, Bridget; AMPATH Kenya<br>Warfa, Osman; Kenya Ministry of Health<br>Dewez, Juan ; London School of Hygiene and Tropical Medicine Faculty of Epidemiology and Public Health<br>Murila, Florence; University of Nairobi School of Medicine<br>Manu, Alexander; Liverpool School of Tropical Medicine<br>Smith, Helen; International Health Consulting Services Ltd<br>Mathai, Matthews; Liverpool School of Tropical Medicine, Centre for Maternal and Newborn Health
Keywords:	NEONATOLOGY, QUALITATIVE RESEARCH, RESPIRATORY MEDICINE (see Thoracic Medicine)

SCHOLARONE™
Manuscripts

I, the Submitting Author has the right to grant and does grant on behalf of all authors of the Work (as defined in the below author licence), an exclusive licence and/or a non-exclusive licence for contributions from authors who are: i) UK Crown employees; ii) where BMJ has agreed a CC-BY licence shall apply, and/or iii) in accordance with the terms applicable for US Federal Government officers or employees acting as part of their official duties; on a worldwide, perpetual, irrevocable, royalty-free basis to BMJ Publishing Group Ltd ("BMJ") its licensees and where the relevant Journal is co-owned by BMJ to the co-owners of the Journal, to publish the Work in this journal and any other BMJ products and to exploit all rights, as set out in our licence.

The Submitting Author accepts and understands that any supply made under these terms is made by BMJ to the Submitting Author unless you are acting as an employee on behalf of your employer or a postgraduate student of an affiliated institution which is paying any applicable article publishing charge ("APC") for Open Access articles. Where the Submitting Author wishes to make the Work available on an Open Access basis (and intends to pay the relevant APC), the terms of reuse of such Open Access shall be governed by a Creative Commons licence – details of these licences and which Creative Commons licence will apply to this Work are set out in our licence referred to above.

Other than as permitted in any relevant BMJ Author's Self Archiving Policies, I confirm this Work has not been accepted for publication elsewhere, is not being considered for publication elsewhere and does not duplicate material already published. I confirm all authors consent to publication of this Work and authorise the granting of this licence.

For peer review only

"Sometimes you are forced to play God…": a qualitative study of healthcare worker experiences of using continuous positive airway pressure in newborn care in Kenya

Helen M Nabwera,[1] Jemma L Wright[2], Manasi Patil[1], Fiona Dickinson[1], Pamela Godia[3], Judith Maua[3], Mercy K. Sammy[4], Bridget C. Naimoi[5], Osman H Warfa[6], Juan E Dewez[7], Florence Murila[8], Alexander Manu[1], Helen Smith[9], Matthews Mathai[1]

1. Centre for Maternal and Newborn Health, Liverpool School of Tropical Medicine, Pembroke Place, Liverpool L3 5QA, United Kingdom
2. Betsi Cadwaladr University Health Board, Wrexham Maelor Hospital, Croesnewydd Road, Wrexham, LL13 7TD
3. Liverpool School of Tropical Medicine, Wilson Business Park, Nairobi, Kenya
4. Gertrude's Children's Hospital, 34 Muthaiga Rd, Nairobi, Kenya
5. AMPATH, Eldoret, Kenya
6. Ministry of Health, Neonatal, Child and Adolescent Health Unit, Afya House, Cathedral Road, P.O. Box 30016–00100, Nairobi
7. London School of Hygiene and Tropical Medicine, Keppel street, London, WC1E 7HT, United Kingdom
8. University of Nairobi, Department of Paediatrics and Child Health & Kenyatta National Hospital, Kenya.
9. Independent consultant, International Health Consulting Services Ltd, Liverpool, United Kingdom.

Corresponding author:

Dr Helen M Nabwera

Liverpool School of Tropical Medicine, Pembroke Place,

Liverpool L3 5QA, United Kingdom

Phone: +44 (0) 151 702 9364

E-mail: helen.nabwera@lstmed.ac.uk

For peer review only - http://bmjopen.bmj.com/site/about/guidelines.xhtml

Abstract

Objective: To explore the experiences of using Continuous Positive Airway Pressure (CPAP) in newborn care among healthcare workers in Kenya, and to identify factors that would promote successful scale-up.

Design and setting: A qualitative study using key informant interviews and focus group discussions, based at secondary and tertiary level hospitals in Kenya.

Participants: Healthcare workers in the Newborn Units providing CPAP.

Primary and secondary outcome measure: Facilitators and barriers of CPAP use in newborn care in Kenya.

Results: 16 key informant interviews and 15 focus group discussions were conducted across 19 hospitals from September 2017 to February 2018. Main barriers reported were: 1) inadequate infrastructure to support the effective delivery of CPAP, 2) shortage of skilled staff rendering it difficult for the available staff to initiate or monitor infants on CPAP, 3) inadequate knowledge and training of staff that inhibited the safe care of infants on CPAP. Key facilitators reported were positive patient outcomes after CPAP use that increased staff confidence and partnership with caregivers in the management of newborns on CPAP. Healthcare workers in private/mission hospitals had more positive experiences of using CPAP in newborn care as the relevant support and infrastructure were available.

Conclusion: CPAP use in newborn care is valued by healthcare workers in Kenya. However, we identified key challenges that threaten its safe use and sustainability.

For peer review only - http://bmjopen.bmj.com/site/about/guidelines.xhtml

Further scale up of CPAP in newborn care should ensure that staff members have ready access to optimal training on CPAP and that there are enough resources and infrastructure to support its use.

Ethics: This study was approved through the appropriate ethics committees in Kenya and the United Kingdom (see in text) with written informed consent for each participant.

For peer review only

Strengths and limitations of this study

- First study in Kenya that has explored the experiences of healthcare workers using CPAP in newborn care in-depth, from the diverse range of services available with different levels of access to resources.
- The study was timely as it was conducted approximately 2 years after an intense implementation programme of CPAP in Newborn Units in public hospitals, therefore providing key information on the sustainability of this exercise.
- The study was conducted at a time when there were several healthcare workers strikes that significantly disrupted health care services in Kenya, therefore the health care worker perspectives in this study may reflect the views of the subset who returned to work after the strikes.

Background

For peer review only - http://bmjopen.bmj.com/site/about/guidelines.xhtml

The neonatal period (i.e. first 28 days after birth) is well recognized globally as the most vulnerable time of childhood [1]. An estimated 2.5 million neonatal deaths occurred worldwide in 2017 accounting for 46% of all under 5 deaths [2]. The majority of these neonatal deaths occurred in low and middle-income countries (LMICs), with the highest burden in South Asia and sub-Saharan Africa [2, 3].

Severe respiratory distress is a serious complication of the three leading causes of neonatal death (i.e. preterm birth, intra-partum related events and sepsis) with a case fatality rate of up to 20% in LMICs [4, 5]. The provision of respiratory support is therefore a key requirement for the care of hospitalised small and sick newborns, but is often inaccessible in many LMICs [4, 6].

Continuous Positive Airway Pressure (CPAP) is a relatively simple and cheap intervention that saves lives, particularly among preterm or very low birth weight neonates (weight<1,500g) with respiratory distress syndrome [6-10]. CPAP has been used in newborn care in high-income countries (HICs) for over 60 years [7]. The use of CPAP and oxygen therapy in HICs was associated with a 75% reduction in neonatal deaths due to respiratory distress syndrome (RDS) [7]. In a trial in Bangladesh, compared to low-flow oxygen therapy alone, bubble CPAP reduced deaths in children under 5 with severe pneumonia and hypoxaemia by 75% [11]. In another trial in Malawi, CPAP reduced deaths by 27% in neonates weighing >1000g with severe respiratory distress [12]. However, CPAP use is also associated with adverse events, such as nasal trauma and pneumothorax, which are rarely reported in LMICs possibly

For peer review only

due to staff limitations in being able to identify them [13-15].

Kenya is one of the 81 countries that account for 90% of child deaths worldwide [16]. Neonatal conditions including severe neonatal infections, birth asphyxia, preterm births and congenital anomalies are the leading cause of under 5 mortality [17]. The neonatal mortality rate in Kenya is higher than the overall global estimate (22 per 1000 live births in 2015) and has reduced slowly over the past few decades [17]. To address the deficiencies in neonatal care, CPAP use in Newborn Units (NBUs) in the public sector has been scaled up since 2014 [18-20]. In addition, the Ministry of Health in Kenya recommended the use of CPAP in newborn care in secondary and tertiary level hospitals and developed a national guideline to support its use in 2016 [21]. However, factors that influence the sustained and effective use of CPAP in newborn care, relevant to the Kenyan context, are largely unknown.

The aim of our study was therefore to explore the experiences of using CPAP in newborn care among healthcare workers, and to identify the barriers and facilitators of its use, thus informing the future scale-up of this life-saving intervention in Kenya.

Methods

Ethics approval

Written informed consent was obtained from each participant prior to enrolment.

For peer review only

For peer review only - http://bmjopen.bmj.com/site/about/guidelines.xhtml

Study Design

This was a qualitative study using key informant interviews (KIIs) and focus group discussions (FGDs). This enabled us to gain an in-depth understanding of the gaps in providing CPAP in newborn care and to identify solutions in the context of a devolved, but resource-constrained public healthcare system, with different governance structures [22-26]. The inclusion of private/mission hospitals also enabled us to gain diverse perspectives on CPAP use in Kenya in the context of differing levels of resources.

Study Population

The study population included all health care workers involved in providing in-patient secondary or tertiary level newborn care services in Kenyan hospitals. The study was conducted across 19 hospitals, involving hospitals within 15 of the 47 counties of Kenya. These included 15 public referral hospitals (8 County, 6 Regional and 1 National level) and 2 private (for-profit) and 2 mission (not-for-profit) hospitals. This constituted 83% of all hospitals in Kenya that provide CPAP newborn care. **(Table 1)**

Setting

Identification of these hospitals was based on information provided by the Division of Child and Adolescent Health at the Ministry of Health of Kenya and non-governmental organisations (NGO's) involved in newborn care in Kenya. A survey that was conducted alongside this study found that across the hospitals, the median number of years of

For peer review only - http://bmjopen.bmj.com/site/about/guidelines.xhtml

CPAP use in newborn care was 3 (IQR 2, 5). The majority (13, 93%) of public hospitals used commercial bubble CPAP acquired predominantly through donor funding, while private/mission hospitals used either ventilator delivered CPAP or 'home-made' bubble CPAP. The median number of CPAP machines per NBU was 2 (IQR 1, 3) and the recommended infrastructure for newborn care and CPAP use was often inadequate in public hospitals [27]. Each NBU was staffed by at least one paediatrician, medical officers, clinical officers, nurses and nursing students. The median number of nurses per shift was 2 (IQR 1, 3) and in an 8-hour shift a nurse cared for a median of 31 (IQR 11, 50) infants [27]. Only 15 (79%) NBUs had clinical guidelines for CPAP use and the majority 11 (58%) had no formal training programmes for medical staff on the use of CPAP [27]. The main indications for initiating CPAP were respiratory distress syndrome in preterm infants and suspected pneumonia [27]. **(Table 2)**

Sampling

Purposive sampling was used to recruit healthcare workers with any experience of working on the NBUs that offered CPAP. This enabled us to get a broad range of views and experiences on CPAP use within and between different NBUs in Kenya [28]. We estimated that at each hospital, a sample size of 10-12 participants would be sufficient to achieve data saturation, where no new themes would emerge from collecting more data from the site [29]. We included all hospitals that provided CPAP in their NBUs and were willing to participate in the study. Our sampling framework is outlined in **Table 3.**

For peer review only - http://bmjopen.bmj.com/site/about/guidelines.xhtml

The FGDs consisted of 6-7 healthcare workers with different levels of expertise in newborn care (including nursing students, nurses, medical officers, clinical officers and senior paediatricians), in order to get a perspective of the teamwork and decision-making process around CPAP use. For the KIIs, we interviewed senior nurses or doctors who we expected to have an appropriate level of knowledge of the operational aspects of CPAP use in their respective NBUs.

Data Collection

Data was collected from September 2017 to February 2018. The KII and FGD guides focused on the following areas in the context of CPAP use in newborn care: training, decision-making for initiating or stopping, resources and interpersonal relationships and was pilot tested with nurses at one of the county referral hospitals. This process and data were used to refine the guides. Informed consent was sought from each participant prior to conducting the FGDs and KIIs. All KIIs and FGDs were conducted face-to-face at the respective hospitals by six research assistants (five female and one male). All research assistants (RAs) were current or former practicing clinicians, all of whom had either used CPAP or were trained in its use. All RAs had received 3-days of intensive training on conducting qualitative interviews/FGDs and were fluent in both English and Kiswahili. All interviews were predominately conducted in English, although some participants used terms in Kiswahili to explain their points. The KIIs/FGDs were recorded and transcribed verbatim by two of the RAs and an independent transcriber. Each of the RAs reviewed the transcripts for accuracy and

For peer review only

discussed these with the lead investigator during debriefing sessions to establish the validity of the transcripts [30].

Data Analysis

We used thematic analysis [26, 31, 32]. Data analysis was an iterative process that started alongside data collection, allowing exploration of key emerging themes [32]. A conceptual framework was used to guide our analysis (**Figure 1**). After extensive familiarization with the data, 5 of the investigators (HN, MP,JW, MKS, BCN) developed a coding scheme, which was revised further during the analysis to ensure the robustness of the approach [33, 34]. NVivo 11 software (QSR International Pty Ltd 2015) was used for data management.

Patient and public involvement statement

This was a countrywide qualitative study that was conducted among health care workers working on Newborn Units in hospitals in Kenya in 2017/2018. This study was designed to explore health care workers experiences of using CPAP in newborn care in Kenya. No patients or members of the public were involved in the design, recruitment or conduct of the study. The results were disseminated to participants through the leadership in their departments and counties, after the study was completed.

Results

We conducted 16 KIIs and 15 FGDs among 100 healthcare workers in Kenya, 77% of

For peer review only - http://bmjopen.bmj.com/site/about/guidelines.xhtml

who were female. The majority were nurses (56%), and in the age range 31-40 years. The overall median number of years of working in health care was 15 years (IQR 8, 17) and in newborn care 4 years (IQR 3, 5). **(Table 4)**

Although healthcare workers felt that the use of CPAP had the potential to enhance newborn care in Kenya, there were multiple challenges with implementation predominantly in the public sector. The key barriers were provision of inadequate infrastructure for effective delivery of CPAP; shortages of skilled staff that hindered CPAP initiation and management; and inadequate knowledge and training of CPAP among staff limiting its safe use. However, these barriers were sometimes mitigated by positive patient outcomes that increased staff confidence in CPAP use and promoting partnership between caregivers (parents or other key family members) and healthcare workers when initiating CPAP. Healthcare workers in better resourced private/mission hospitals had more positive experiences of using CPAP in newborn care.

Effect of inadequate infrastructure on optimal delivery of CPAP

Overall, the infrastructure to support CPAP use in newborn care within the public sector was reported as inadequate. Healthcare workers highlighted that there were often not enough CPAP machines to meet patient demands, meaning that they frequently faced the difficult decision of rationing CPAP.

"Sometimes you are forced to play God, which we don't like. We would want to have as many CPAP machines as possible but, yeah, it's not possible. So, this is the matter

For peer review only - http://bmjopen.bmj.com/site/about/guidelines.xhtml

of weighing and looking at who is most likely to survive." (KII, Paediatrician NBU 5)

Staff described the ethical dilemma of discontinuing CPAP support inappropriately early in a baby that was improving in order to initiate it on a sicker baby. This was extremely distressing for staff.

"It is a psychological torture to us and even to them, to the mother. So, such experiences we have had them severally and even for us we feel guilty "If I could have done this for the baby they could have survived as these ones", you see" (FGD, NBU 8, Nurse)

There were also frequent shortages of key CPAP consumables in NBUs, such as appropriately sized nasal prongs, due to difficulties with procurement. Healthcare workers reported that after the completion of donor-supported implementation, hospitals struggled to find affordable suppliers for these consumables. The NBUs therefore either improvised or reused equipment that was supposed to be disposed of, posing an infection risk to the newborn and increasing the risk of other adverse events.

" Like the nasal prongs size zero is usually a challenge…sometimes they go out of stock…we usually wash them, they are actually supposed to be disposed but we are allowed to wash them even if they are not visibly dirty, but even if you washed them, it reaches that time you must throw them." (FGD, NBU 8, Nurse)

General infrastructure difficulties also meant that use of CPAP was restricted by other

For peer review only - http://bmjopen.bmj.com/site/about/guidelines.xhtml

issues, such as unreliable power and oxygen supplies, and lack of physical space in the NBUs for the CPAP machinery.

"At times our electricity is on and off, I remember a time a baby was on CPAP and our generator had a problem and we were told to wait for around thirty-five minutes for them to fuel the generator. So, it interfered with that process." (FGD, NBU 14, Nurse)

"Another factor is the availability of space, like in our nursery when you come when we have babies, we just put babies close, …and CPAP needs a spacious place. So sometimes you can have a challenge starting it, because where will you put these babies so that you make a space for that child." (FGD, NBU 8, Nurse)

The data also highlighted a lack of other relevant resources required to treat neonates, specifically neonatal medications and laboratory support.

"Again, the other challenge is the laboratory investigations, the baby is on CPAP, maybe you will need investigation like blood cultures, but you find that the hospital does not have enough reagents… So, this is a baby on CPAP but you cannot even investigate" (FGD, NBU 9 Nurse)

Shortages of skilled staff that hindered CPAP initiation and management

Staffing issues across most NBUs were identified as a key barrier to the use of CPAP, either due to staff shortages, a high staff turnover or healthcare worker strikes. It was highlighted that babies on CPAP need extra monitoring by the nursing staff, so staff shortages were felt to directly limit CPAP use on the NBUs. Experiences included times

For peer review only - http://bmjopen.bmj.com/site/about/guidelines.xhtml

on busy shifts when the staff did not to put babies on CPAP, using oxygen due to the extra monitoring required for babies on CPAP, even though they knew this would result in suboptimal care for the baby.

"You find you are only two with more than twenty babies in the NBU, you feel if I fix (CPAP), I am still doing other things and I will not be able to observe the baby as required" (FGD, NBU 6, Nurse)

Inadequate numbers of staff with skills for using CPAP also hindered staff from initiating this therapy, and healthcare workers reported that many of them had not received training on using CPAP. As a result, some reported witnessing inappropriate cessation of CPAP when a patient was handed over to other member of staff.

"So, if I'm working in the morning, I would be able to use CPAP. There is the other person who comes by night. If they feel they are not comfortable in the use of CPAP, they wouldn't put children on CPAP." (KII, NBU 5)

Unfortunately, there were several prolonged healthcare providers' strikes in the public sector during our data collection, which disrupted CPAP use. These strikes resulted in reduced confidence in using CPAP amongst healthcare providers, many of who had received in-house training but were dependent on resources or senior support lost during the strikes.

"We have the cards (containing instructions for setting up CPAP)…With the strike right now I don't know exactly where they are but I think we are going to find them

For peer review only

For peer review only - http://bmjopen.bmj.com/site/about/guidelines.xhtml

somehow." (FGD, NBU 15, Nurse)

Inadequate knowledge and training for appropriate CPAP use

The introduction of CPAP to the public sector NBUs often occurred with support of NGOs. The acquisition of CPAP machines was accompanied by initial training of the senior members of staff in each unit, with a plan to cascade this training to other staff members during in-house educational meetings.

"I think one of the things that has made it work in this hospital is the good training that has been passed on from the pioneer group that was trained." (FGD, NBU 17, Medical Officer)

However inadequate training of staff was reported in most of the NBUs, as the plan to cascade the training often did not occur. In some cases, the most senior members of the team with skills for using CPAP left and were replaced with new staff members untrained in the use of CPAP. This resulted in a decline in the skills and confidence of using CPAP among healthcare workers in these NBUs to the point that CPAP was no longer used even though the equipment was available and in working order.

"Just after the training, I think our paediatrician left the facility and from that time, utilization and prescription of CPAP was very, very minimal and kept deteriorating until now it is like nobody is thinking about CPAP" (FGD, NBU 6, Senior nurse)

On-going training on CPAP was variable with staff from private/mission hospitals describing regular and structured training, whilst most public sector healthcare

For peer review only

For peer review only - http://bmjopen.bmj.com/site/about/guidelines.xhtml

workers only received informal on-the-job training. This training was very dependent on the level of support for CPAP within the unit and the presence of key educators. From our data we found that training occurred more regularly in private and mission hospitals, where there was access to better resources and fewer admissions.

"Once they get the training, they are very positive and also willing to train others. So, it does not matter whoever is training, so long as you have been trained by somebody else it is something you pass to the next person." (FGD, NBU 17, Senior nurse)

However, even with regular training, the frequent rotation of staff between hospital departments was also noted to have a significant impact on the competencies of the staff.

"One of the biggest challenge is the structure of the healthcare system because the people we started with, the ones who were trained were changed, and a new team comes which is totally not competent and you tell them about CPAP and they fear." (KII, NBU 9)

The healthcare workers also had limited knowledge about the identification and management of common CPAP complications, resulting in potentially unsafe use of CPAP.

"I will not deny because I have not been taught how to identify complications on CPAP." (FGD, NBU 14, Medical Officer)

Notably, the presence of a 'CPAP champion' in an NBU was noted to improve the

For peer review only

frequency of training and level of knowledge about CPAP among the staff members. Good leadership was critical in ensuring that all staff members were trained to use CPAP.

"We have 2 champion nurses, we have the nursing officer in charge…she got to understand how they connect, and what are the essential things with the CPAP, then now what she does is she trains any new nurses who come through." (KII, NBU 12)

Positive infant outcomes that increased staff confidence in CPAP use

Staff in NBUs felt that CPAP was useful in improving neonatal care when used appropriately. Indeed, personal experience of good outcomes associated with CPAP use was identified as improving staff morale and their confidence in using CPAP.

"There are some babies we had managed there who had (a) bad prognosis and sometimes you put (them) on CPAP and you see the baby is not in (a) very good state. As I have said the prognosis is bad, but you put on CPAP and you monitor, and they pull through. Even other healthcare providers, they get surprised" (FGD, NBU 14, Medical Officer)

However, there was recognition that when it was used inappropriately, for example when the recommended criteria for initiating or stopping CPAP were not adhered to, the outcomes were poor. This resulted in diminished confidence of CPAP use among staff and had a negative impact on its sustainability in those units.

"It has instilled fear among the nurses such that you take them through the training

For peer review only

For peer review only - http://bmjopen.bmj.com/site/about/guidelines.xhtml

but when you tell them to go and start, you find that they fear because they don't want to be associated with starting a CPAP and a baby dying, that is now the problem." (FGD, NBU 9, Nurse)

High staff confidence and engagement was identified as important in promoting the use of CPAP. The empowerment of all members of the clinical team to initiate CPAP on newborns who meet the criteria was important in promoting its use, particularly in reducing delays in starting this intervention. This was especially noted if staff members were well supported by the leadership of the hospital.

"The CPAP is good because the nurse can assess prescribe and administer even without a doctor and when the outcome is so good, it's motivating to the nurses. When you work like that you get so excited you feel you're empowered, and you feel like doing it more and more times." (FGD, NBU 8, Nurse)

Effect of partnerships between caregivers and healthcare workers on uptake of CPAP

Use of appropriate communication skills that involved the caregivers in the decision-making of care strategies for their infants was felt to enhance acceptability of CPAP.

"Our policy is that they really have to consent, and not just consent in feeling that you are coercing them...Our target is to end up with a win-win, have a mutual agreement. It makes our work easier, and makes the baby get well faster. Because we are working as a team" (FG, NBU 1, Nurse)

For peer review only

For peer review only - http://bmjopen.bmj.com/site/about/guidelines.xhtml

Caregivers were also reported to be more receptive to the use of CPAP if they were actively encouraged to be involved in the care of their baby whilst on CPAP.

‘The mother or the caregiver must be aware, you must counsel them, they accept before you start CPAP because if they refuse then it means they’ll interfere with the technical bit of it.” (FGD, NBU 8, Nurse)

Overall, in units where the outcomes of the babies on CPAP were good, the healthcare workers reported that both the main caregivers and the wider community became more receptive to CPAP.

“It’s a bit tricky for ... if you put CPAP and a child passes on, nobody will now accept CPAP, no one will accept. If the one improves, the next one also wants it”. (FGD, NBU 5, Nurse)

Discussion

Our study describes experiences of healthcare workers using CPAP within NBUs in Kenya. The implementation gaps were predominantly in the public sector and these were mainly at the health system level. However, we also found factors at a personal level for both caregivers and health care providers that facilitated CPAP use, even in the context of resource limitations.

Our study found that CPAP was felt to be beneficial when the right training and infrastructure was in place for its use. This is consistent with findings from an observational study in a rural Ugandan neonatal intensive care unit (NICU), which

For peer review only

found that training using the Silverman-Anderson respiratory severity score made it feasible to implement CPAP in newborn care safely in a resource-limited setting [35]. Another study in a rural mission hospital in Kenya showed higher survival-to-discharge rates in premature infants with severe respiratory distress syndrome after the introduction of CPAP [19]. In Malawi, a non-randomized controlled study also showed a 27% absolute improvement in survival of neonates with severe RDS [12] and that CPAP was a cost-effective strategy for the care of newborns with respiratory difficulty when compared to nasal oxygen therapy [36]. However, these studies were all small scale, supported by external funding. In the absence of such external funding, our data suggests that poor infrastructure is a major hindrance to the safe and effective use of CPAP in Kenyan public hospitals. A recent study from India also reported the impact of resource constraints on the sustainability and safe implementation of CPAP in newborn care [37].

The inadequate number of skilled staff, particularly nursing staff, was a major challenge. Indeed, nurses within our study reported being reluctant to start CPAP use due to poor nurse: patient ratios leading to the inability to monitor these babies adequately while on CPAP- a WHO prerequisite for using CPAP [8, 38]. Inadequate staffing of NBUs is a major challenge in Kenya, with the current median nurse: baby ratio in the public sector reported to be as high as 17 babies per nurse [39]. Recent exploratory work in hospitals in Nairobi, Kenya found that nurses had developed strategies to cope with their workload by task-shifting, whereby less technical tasks are shifted to student nurses, other support staff or mothers to free-up nursing time

For peer review only - http://bmjopen.bmj.com/site/about/guidelines.xhtml

to focus on the more technical aspects of newborn care [40]. Future projects are planned that will seek to formalise this task-shifting to support nurses working in these busy NBUs that may also have an impact on the delivery of CPAP [40].

Inadequate training of healthcare workers was another common problem identified within our data that had a negative impact on CPAP use. A recent cross-sectional study from Kenya also found that over a third of sick newborns were cared for in an environment where nursing knowledge was very low [41]. When CPAP was introduced in public hospitals in western Kenya, a successful training-of-trainers model was used [18], but our findings suggest that its effectiveness was not sustained over time. Regular staff rotations and prolonged healthcare worker strikes in the public sector appears to have had a negative impact on CPAP related competencies of staff. In the context of limited training opportunities, work has been done in Malawi to develop a simple clinical algorithm, to identify newborns who would benefit most from CPAP, with evidence of high inter-rater reliability, sensitivity and specificity for their new 'TRY' tool [42, 43]. In addition, new training-of-trainers programs [44] and health partnership models where international experts support training strategies over a longer period of time [45], could be beneficial within Kenya. Sustained refresher training on the use of CPAP in newborn care units in LMICs is also crucial. Indeed, a recent retrospective cohort study in rural Rwanda that found that even after training staff, identification of eligible newborns was a challenge [46].

Finally, our findings also suggest that partnership between healthcare workers and

For peer review only - http://bmjopen.bmj.com/site/about/guidelines.xhtml

the main caregivers in the decision-making surrounding CPAP use in their babies is critical to the acceptability of this intervention. Internationally, there is a growing recognition of the importance of involving parents in the care of their hospitalized newborns in improving outcomes for both the infant and their family [47]. A descriptive study conducted at a tertiary newborn unit in Malawi found that caregivers felt anxious and fearful when their infants were on CPAP with limited parent-child interaction [48]. However, this anxiety was mitigated by support from healthcare providers, family and friends [48]. In Rwanda a 'parental neonatal curriculum' has recently been developed using a Delphi consensus technique involving parents and healthcare workers [49]. The development of such curricula has the potential to enhance true partnership with parents [49]. In Malawi, Family-Led Care Models have recently been implemented to improve the facility and home-based care of preterm/low birth weight newborns [50]. The evaluation of the impact of these strategies is keenly awaited.

The key limitation of this study was that it was conducted at a time when there were several healthcare workers strikes that significantly disrupted health care services in Kenya. Our findings may therefore reflect the views predominantly of healthcare workers who returned to work after the strikes.

Nevertheless, to our knowledge, this is the first study in Kenya that has explored in-depth the experiences of healthcare workers using CPAP in newborn care. Our study involved healthcare workers in both the public and private/mission sectors of Kenya,

For peer review only - http://bmjopen.bmj.com/site/about/guidelines.xhtml

enabling us to identify a diverse range of perspectives from healthcare professionals within the same country but with access to different levels of resources for providing newborn care. These findings will be pertinent for the future scale up strategies for CPAP and other newborn care technologies in Kenya and other similar settings.

Conclusion

The scale-up of CPAP use in newborn care in Kenya is a strategy that healthcare workers value as a key intervention for improving the survival of newborns. However, there are important challenges that need to be addressed to ensure sustainability and safety of its use in Kenyan hospitals. Worryingly, newborns are currently exposed to preventable risks, limiting the benefit of CPAP in these vulnerable babies. Future CPAP implementation strategies should ensure that infrastructure for CPAP use is fit for purpose, shortages of skilled staff are addressed, and that staff training and partnership with caregivers are prioritised.

Acknowledgements

We would like to thank the health care workers at the Newborn Units of the following hospitals: Aga Khan University Hospital, Nairobi; Bungoma County Referral Hospital; Busia County Referral Hospital; Coast General Hospital, Mombasa; Gertrude's Children's Hospital, Nairobi; Homabay County Referral Hospital; Jaramogi Oginga Odinga Teaching and Referral Hospital, Kisumu; Kakamega County Referral Hospital; Kenyatta National Hospital; Kiambu County Hospital; Kijabe Mission Hospital; Kisii County Referral Hospital; Kisumu County Referral Hospital; Machakos County Referral

For peer review only - http://bmjopen.bmj.com/site/about/guidelines.xhtml

Hospital; Mama Lucy Hospital, Nairobi; Migori County Referral Hospital; Naivasha Sub-county Hospital; Pumwani Maternity Hospital, Nairobi; Siaya County Referral Hospital; Tenwek Mission Hospital, Bomet. We are grateful also to country health representatives and the Ministry of Health in Kenya for their participation and support in conducting this study. Many thanks to our colleagues Ms Joyce Mutuku, Mr Onesimus Muchemi, Ms Sylvia Gichuru, Ms Linda Omolo, Ms Beatrice Ochieng who supported us with data collection. We thank Dr Sarah White, Ms Sophie Ngugi and Mr Allan Govoga for their important contributions to the conceptualization of this study. We thank Dr Bernard Olayo and Miss Caroline Kendi (Centre for Public Health and Development, Kenya) for their advice and support.

Contributors

HMN coordinated the study, analyzed the data, prepared the manuscript and approved the final manuscript as submitted. She took over as Principal Investigator of the study prior to the commencement of data collection and coordinated all aspects of the project thereafter. JW, MP analyzed the data and prepared the manuscript and approved the final manuscript as submitted. FD led the data management of this study, reviewed and approved the final manuscript as submitted. She was the UK-based Research Assistant in this study. PG conceptualised the study, reviewed and approved the and approved the final manuscript as submitted. She led with the coordination of the ethics approval processes in Kenya. JM conceptualised the study, provided support with the coordination of study activities, reviewed and approved the

For peer review only

final manuscript as submitted. She led the communication strategies with the Kenyan stakeholders. MKS and BCN collected, transcribed and analyzed the data. They were Research Assistants in this study in Kenya. OHW conceptualised the study, reviewed and approved the final manuscript as submitted. He facilitated the approval of study activities at the national and facility level. JED conceptualised the study, reviewed and approved the and approved the final manuscript as submitted. He was the initial Principal Investigator of the study and led the ethics application process. FM conceptualised the study, reviewed and approved the final manuscript as submitted. She was co-Principal Investigator and supported the ethics application processes of the study in Kenya. She also provided very valuable context-relevant information during the data collection and analysis phases. AM supported with study coordination, reviewed and approved the final manuscript as submitted. HS provided guidance on the qualitative methodology during the conceptualization of the study, reviewed and approved the and approved the final manuscript as submitted. She was the lead Social Scientist for this study and provided critical review of the data collection and analysis. MM conceptualised the study, reviewed and approved the and approved the final manuscript as submitted. He provided oversight for all the study processes.

Funding

This project was funded by the UK Department for International Development (Project number: 202549), awarded to Prof Nynke van den Broek.

Competing interests

For peer review only - http://bmjopen.bmj.com/site/about/guidelines.xhtml

None declared.

Patient consent

Not required.

Ethics approval

The study was approved by the Research and Ethics Committee at the Liverpool School of Tropical Medicine (protocol number:15-032) and the University of Nairobi-Kenyatta National Hospital Ethics Research Committee (P56/02/2017).

Provenance and peer review

Not commissioned; externally peer reviewed.

Data sharing statement

All data relevant to the study are included in the article. Extra data is available by emailing matthews.mathai@lstmed.ac.uk.

Open access

This is an open access article distributed in accordance with the Creative Commons Attribution Non Commercial (CC BY-NC 4.0) license, which permits others to distribute, remix, adapt, build upon this work non-commercially, and license their derivative works on different terms, provided the original work is properly cited, appropriate credit is given, any changes made indicated, and the use is non-

For peer review only - http://bmjopen.bmj.com/site/about/guidelines.xhtml

commercial. See: http://creativecommons.org/licenses/by-nc/4.0/.

For peer review only

References

1. **Neonatal mortality** [https://data.unicef.org/topic/child-survival/neonatal-mortality/]
2. UN-IGME.: **Levels and Trenda in Child Mortality**. In. New York; 2018.
3. Lawn JE, Blencowe H, Oza S, You D, Lee AC, Waiswa P, Lalli M, Bhutta Z, Barros AJ, Christian P *et al*: **Every Newborn: progress, priorities, and potential beyond survival**. *Lancet* 2014, **384**(9938):189-205.
4. Duke T: **CPAP and high-flow oxygen to address high mortality of very severe pneumonia in low-income countries - keeping it in perspective**. *Paediatr Int Child Health* 2019, **39**(3):155-159.
5. UN-IGME.: **Levels and trends in child mortality: Report 2015**. In. New York, USA: UN-Interagency group for child mortality estimation; 2017.
6. Duke T: **CPAP: a guide for clinicians in developing countries**. *Paediatr Int Child Health* 2014, **34**(1):3-11.
7. Ho JJ, Subramaniam P, Davis PG: **Continuous distending pressure for respiratory distress in preterm infants**. *Cochrane Database Syst Rev* 2015(7):CD002271.
8. WHO: **WHO recommendation on continuous positive airway pressure therapy for the treatment of preterm newborns with respiratory distress syndrome**. In. Geneva: WHO; 2015.
9. Okello F, Egiru E, Ikiror J, Acom L, Loe K, Olupot-Olupot P, Burgoine K: **Reducing preterm mortality in eastern Uganda: the impact of introducing low-cost bubble CPAP on neonates <1500 g**. *BMC Pediatr* 2019, **19**(1):311.
10. Ezenwa B, Akintan P, Fajolu I, Ladele J, Ezeaka C: **Bubble CPAP in the Management of Respiratory Distress Syndrome in Resource Constrained Settings: The Luth Experience.** *Pediatr Oncall J* 2016, **13**:9-12.
11. Chisti MJ, Salam MA, Smith JH, Ahmed T, Pietroni MA, Shahunja KM, Shahid AS, Faruque AS, Ashraf H, Bardhan PK *et al*: **Bubble continuous positive airway pressure for children with severe pneumonia and hypoxaemia in Bangladesh: an open, randomised controlled trial**. *Lancet* 2015, **386**(9998):1057-1065.
12. Kawaza K, Machen HE, Brown J, Mwanza Z, Iniguez S, Gest A, Smith EO, Oden M, Richards-Kortum RR, Molyneux E: **Efficacy of a low-cost bubble CPAP system in treatment of respiratory distress in a neonatal ward in Malawi**. *PLoS One* 2014, **9**(1):e86327.
13. Lissauer T, Duke T, Mellor K, Molyneux L: **Nasal CPAP for neonatal respiratory support in low and middle-income countries**. *Arch Dis Child Fetal Neonatal Ed* 2017, **102**(3):F194-F196.
14. Dewez JE, van den Broek N: **Continuous positive airway pressure (CPAP) to treat respiratory distress in newborns in low- and middle-income countries**. *Trop Doct* 2017, **47**(1):19-22.
15. Fischer C, Bertelle V, Hohlfeld J, Forcada-Guex M, Stadelmann-Diaw C, Tolsa JF: **Nasal trauma due to continuous positive airway pressure in neonates**. *Arch Dis Child Fetal Neonatal Ed* 2010, **95**(6):F447-451.
16. Countdown to C: **Countdown to 2030: tracking progress towards universal coverage for reproductive, maternal, newborn, and child health**. *Lancet* 2018, **391**(10129):1538-1548.
17. **Kenya: neonatal and child health profile**

For peer review only - http://bmjopen.bmj.com/site/about/guidelines.xhtml

[https://www.who.int/pmnch/media/membernews/2011/20121216_kenyaparliament.pdf Accessed 12 June 2019]
18. Olayo B, Kirigia CK, Oliwa JN, Agai ON, Morris M, Benckert M, Adudans S, Murila F, Wilson PT: **Effective training-of-trainers model for the introduction of continuous positive airway pressure for neonatal and paediatric patients in Kenya**. *Paediatr Int Child Health* 2019, **39**(3):193-200.
19. Myhre J, Immaculate M, Okeyo B, Anand M, Omoding A, Myhre L, Okeyo L, Barasa I, Letchford S: **Effect of Treatment of Premature Infants with Respiratory Distress Using Low-cost Bubble CPAP in a Rural African Hospital**. *J Trop Pediatr* 2016, **62**(5):385-389.
20. Murphy GAV, Omondi GB, Gathara D, Abuya N, Mwachiro J, Kuria R, Tallam-Kimaiyo E, English M: **Expectations for nursing care in newborn units in Kenya: moving from implicit to explicit standards**. *BMJ Glob Health* 2018, **3**(2):e000645.
21. **Basic Paediatric Protocols** [https://www.psk.or.ke/public/uploads/file/c0d3675787d651dedbf4a0edfc9a2898.pdf Accessed 19 August 2019]
22. Pyone T, Smith H, van den Broek N: **Implementation of the free maternity services policy and its implications for health system governance in Kenya**. *BMJ Glob Health* 2017, **2**(4):e000249.
23. McCollum R, Limato R, Otiso L, Theobald S, Taegtmeyer M: **Health system governance following devolution: comparing experiences of decentralisation in Kenya and Indonesia**. *BMJ Glob Health* 2018, **3**(5):e000939.
24. McCollum R, Taegtmeyer M, Otiso L, Muturi N, Barasa E, Molyneux S, Martineau T, Theobald S: **"Sometimes it is difficult for us to stand up and change this": an analysis of power within priority-setting for health following devolution in Kenya**. *BMC Health Serv Res* 2018, **18**(1):906.
25. Al-Busaidi ZQ: **Qualitative research and its uses in health care**. *Sultan Qaboos Univ Med J* 2008, **8**(1):11-19.
26. Patton M: **Qualitative evaluation and research methods**. Beverly Hills, C.A.: Sage; 1990.
27. Liverpool School of Tropical Medicine, Department of International Development, Ministry of Health Kenya: **Assessment of the use of Continuous Positive Airway Pressure (CPAP) in newborn care in Kenya**. In.: Liverpool School of Tropical Medicine; 2019.
28. Mays N, Pope C: **Qualitative research in health care. Assessing quality in qualitative research.** *BMJ* 2000, **320**(7226):50-52.
29. Guest G, Bunce A, Johnson L: **How Many Interviews Are Enough? An Experiment with Data Saturation and Variability**. *Field Methods* 2006, **18**(1):59-82.
30. Kvale S: **An introduction to qualitative research**: SAGE; 1996.
31. Pope C, Ziebland S, Mays N: **Qualitative research in health care. Analysing qualitative data**. *BMJ* 2000, **320**(7227):114-116.
32. Braun V, Clarke V: **Using thematic analysis in psychology**. *Qualitative Research in Psychology* 2006, **3**(2):77-101.
33. Tuckett AG: **Applying thematic analysis theory to practice: A researcher's experience.** *Contemporary Nurse* 2005, **19**(1-2):75-87.
34. Carey JW, Morgan M, Oxtoby MJ: **Intercoder Agreement in Analysis of Responses to Open-Ended Interview Questions: Examples from Tuberculosis Research** *Cultural Anthropology Methods* 1996, **8**(3):1-5.

For peer review only - http://bmjopen.bmj.com/site/about/guidelines.xhtml

35. McAdams RM, Hedstrom AB, DiBlasi RM, Mant JE, Nyonyintono J, Otai CD, Lester DA, Batra M: **Implementation of Bubble CPAP in a Rural Ugandan Neonatal ICU**. *Respir Care* 2015, **60**(3):437-445.
36. Chen A, Deshmukh AA, Richards-Kortum R, Molyneux E, Kawaza K, Cantor SB: **Cost-effectiveness analysis of a low-cost bubble CPAP device in providing ventilatory support for neonates in Malawi - a preliminary report**. *BMC Pediatr* 2014, **14**:288.
37. Dewez JE, Chellani H, Nangia S, Metsis K, Smith H, Mathai M, van den Broek N: **Healthcare workers' views on the use of continuous positive airway pressure (CPAP) in neonates: a qualitative study in Andhra Pradesh, India**. *BMC Pediatr* 2018, **18**(1):347.
38. WHO: **WHO recommendations on interventions to improve preterm birth outcomes**. In. Geneva: WHO; 2015.
39. Gathara D, Serem G, Murphy GAV, Obengo A, Tallam E, Jackson D, Brownie S, English M: **Missed nursing care in newborn units: a cross-sectional direct observational study**. *BMJ Qual Saf* 2019.
40. Nzinga J, McKnight J, Jepkosgei J, English M: **Exploring the space for task shifting to support nursing on neonatal wards in Kenyan public hospitals**. *Hum Resour Health* 2019, **17**(1):18.
41. Murphy GAV, Gathara D, Mwaniki A, Nabea G, Mwachiro J, Abuya N, English M: **Nursing knowledge of essential maternal and newborn care in a high-mortality urban African setting: A cross-sectional study**. *J Clin Nurs* 2019, **28**(5-6):882-893.
42. Hundalani SG, Richards-Kortum R, Oden M, Kawaza K, Gest A, Molyneux E: **Development and validation of a simple algorithm for initiation of CPAP in neonates with respiratory distress in Malawi**. *Arch Dis Child Fetal Neonatal Ed* 2015, **100**(4):F332-336.
43. Crehan C, Colbourn T, Heys M, Molyneux E: **Evaluation of 'TRY': an algorithm for neonatal continuous positive airways pressure in low-income settings**. *Arch Dis Child* 2018, **103**(8):732-738.
44. Wilson PT, Benckert MM, Moresky RT, Morris MC: **Development and Implementation of a Training-of-Trainers Program for Continuous Positive Airway Pressure in Neonatal and Pediatric Patients in Five Low- and Middle-Income Countries**. *J Trop Pediatr* 2017, **63**(5):358-364.
45. Ntigurirwa P, Mellor K, Langer D, Evans M, Robertson E, Tuyisenge L, Groves A, Lissauer T: **A health partnership to reduce neonatal mortality in four hospitals in Rwanda**. *Global Health* 2017, **13**(1):28.
46. Nahimana E, Ngendahayo M, Magge H, Odhiambo J, Amoroso CL, Muhirwa E, Uwilingiyemungu JN, Nkikabahizi F, Habimana R, Hedt-Gauthier BL: **Bubble CPAP to support preterm infants in rural Rwanda: a retrospective cohort study**. *BMC Pediatr* 2015, **15**:135.
47. Banerjee J, Aloysius A, Platonos K, Deierl A: **Family centred care and family delivered care – What are we talking about?** *J of Neonatal Nursing* 2018, **24**(1):8-12.
48. Gondwe MJ, Gombachika B, Majamanda MD: **Experiences of caregivers of infants who have been on bubble continuous positive airway pressure at Queen Elizabeth Central Hospital, Malawi: A descriptive qualitative study**. *Malawi Med J* 2017, **29**(1):10-15.
49. Musabyemungu JA, Willson A, Batenhorst S, Webbe J, Cartledge PT: **What topics should we teach the parents of admitted neonates in the newborn care unit in the resource-limited setting - a Delphi study**. *Matern Health Neonatol Perinatol* 2019,

For peer review only - http://bmjopen.bmj.com/site/about/guidelines.xhtml

5:11.
50. **Every Preemie scale** [https://www.everypreemie.org/malawi/family-led-care Accessed 13 August 2019]

For peer review only

TABLES

Table 1: List of Newborn Baby Units with CPAP by County

Name of County	Population of County (millions)	Number of Newborn Baby Units using CPAP
Nairobi	3.1	6
Kiambu	1.6	2
Machakos	1.1	1
Nakuru	1.6	2
Mombasa	0.9	1
Uasin Gishu	0.9	1
Bomet	0.7	1
Kisumu	0.3	2
Homa Bay	0.9	1
Siaya	0.8	1
Migori	0.9	1
Kisii	0.2	1
Bungoma	1.4	1
Kakamega	1.7	1
Busia	0.7	1
Total		23

Table 2: Characteristics of the hospitals in relation to CPAP implementation

	National referral hospital (Level 6) N=1	Regional referral hospital (Level 5) N=6	County referral hospital (Level 4) N=8	Private N=2	Mission N=2	Overall N=19
Number of years of CPAP use in newborn care, median (IQR)	4	3 (3, 3)	3 (2.5, 3)	9 (6,12)	11.5 (7, 16)	3 (2, 5)
Number of CPAP machines per newborn care unit, median (IQR)	10	2 (1, 3)	1 (1, 2)	4 (2, 6)	8(8, 8))	2 (1, 3)
Emergency power supply present, n (%)	1 (100)	6 (100)	8 (100)	2 (100)	2(100)	19 (100)
Emergency power always works, n (%)	1 (100)	6 (100)	4 (50)	2 (100)	2 (100)	15 (79)
Commercial bubble CPAP, n (%)	-	6 (100)	7 (88)	0	2 (100)	13 (68)
Maximum number of newborns admitted at any one time, median	160 (160, 160)	56 (30, 85)	24 (12, 38)	7 (6, 7)	36 (21, 50)	31 (11, 50)

For peer review only

For peer review only - http://bmjopen.bmj.com/site/about/guidelines.xhtml

**(IQR)**						
**Number of nurses per shift, median (IQR)**	18 (18, 18)	2 (1, 3)	1 (1, 2)	4 (4, 4)	3 (3, 4)	2 (1, 3)
**Number of babies on CPAP per nurse, median (IQR)**	3 (3, 3)	1 (1, 2)	1 (1, 3)	2 (2, 2)	2 (2, 2)	2 (1, 2)
**Pre-defined CPAP training plan for medical staff, n (%)**	1 (100)	4 (67)	2 (25)	1 (50)	0	8 (42)
**CPAP trained nurse on every shift, n (%)**	1 (100)	3 (50)	1 (13)	1 (50)	0	6 (32)
**CPAP trained doctor/ clinical officer on 24hour shift, n (%)**	1 (100)	3 (50)	1 (13)*	1 (50)	0	6 (32)*
**Nurse reported to be competent in neonatal resuscitation per shift, n (%)**	1 (100)	6 (100)	6 (75)	2 (100)	2 (100)	17 (89)
**Clinical guidelines for CPAP, n (%)**	1 (100)	5 (83)	7 (88)	1 (50)	1 (50)	15 (79)

Table 3: Sampling Framework

**Number of Hospitals using CPAP in newborn care**	23		
**Type of Hospital**	18 Public	3 Private or 2 Mission	
**Hospitals in Study**	15<br>Public	2<br>private	2<br>mission

For peer review only - http://bmjopen.bmj.com/site/about/guidelines.xhtml

Table 4: Characteristics of study participants

	National referral hospital (Level 6) N=8	Regional referral hospital (Level 5) N=36	County referral hospital (Level 4) N=36	Private N=8	Mission N=12	Overall N=100
**Majority age group, years**	31-40, 41-50	18-30	31-40	31-40	18-30	31-40
**Females, n (%)**	7 (88)	26 (72)	27 (77)	8 (100)	9 (75)	77 (77)
**Staff cadre**						
**Medical students, n**	1					1
**Nurses/midwives, n**	5	20	19	6	6	56
**Clinical officers, n**		4	1		1	6
**Medical officers, n**	3	9	11	1	3	27
**Paediatricians, n**		3	4	1	2	10
**Years of experience in health care, median (IQR)**	17 (9, 23)	15 (4, 23)	8 (2, 20)	17 (12, 19)	2 (2, 6)	15 (8, 17)
**Years of experience in newborn care, median (IQR)**	4 (3, 10)	5 (2, 10)	3 (1, 6)	5 (4, 7)	2 (1, 4)	4 (3, 5)

For peer review only

FIGURE LEGEND

Figure 1: Conceptual framework used to guide data analysis

For peer review only

For peer review only - http://bmjopen.bmj.com/site/about/guidelines.xhtml

Figure 1: Conceptual Framework

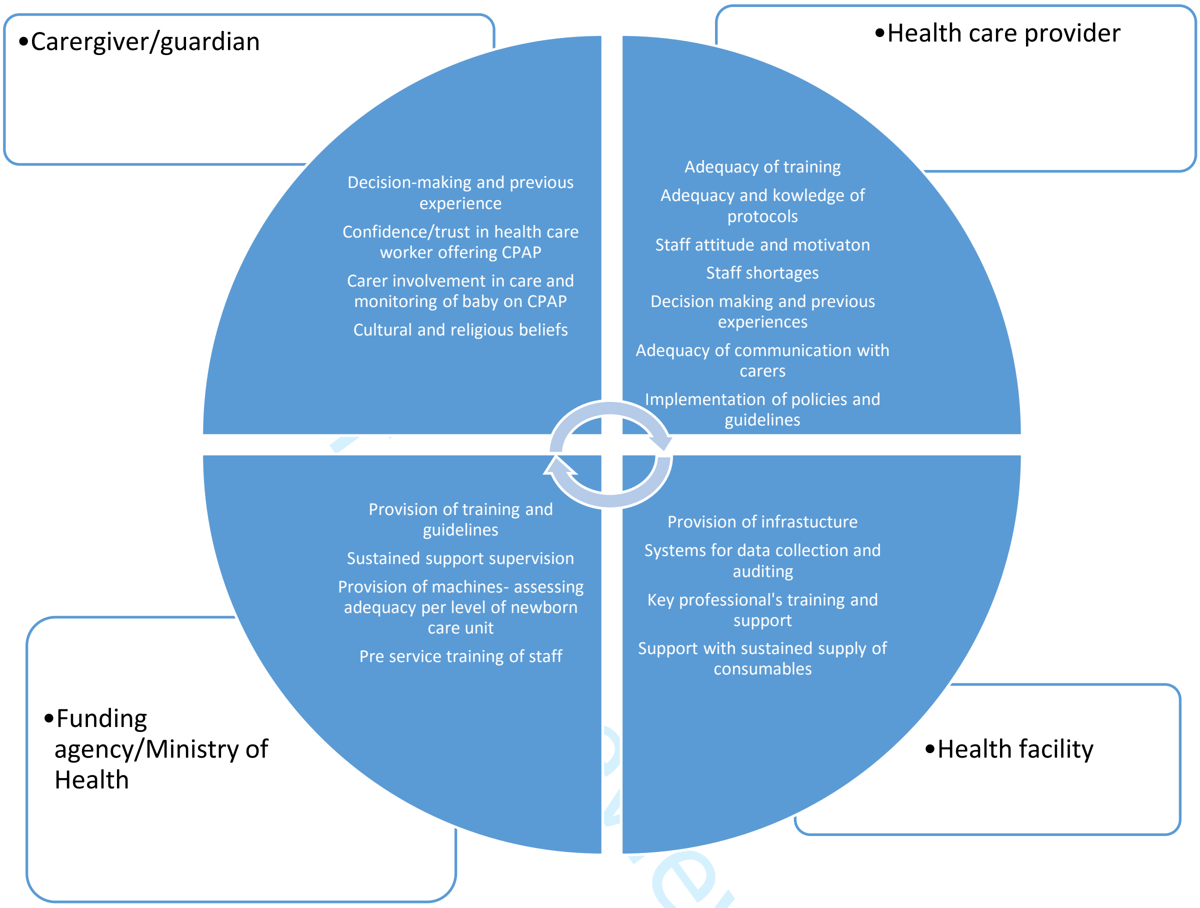

BMJ Open

“Sometimes you are forced to play God...”: a qualitative study of healthcare worker experiences of using continuous positive airway pressure in newborn care in Kenya.

Journal:	*BMJ Open*
Manuscript ID	bmjopen-2019-034668.R1
Article Type:	Original research
Date Submitted by the Author:	19-Mar-2020
Complete List of Authors:	Nabwera, Helen; Liverpool School of Tropical Medicine, International Public Health<br>Wright, Jemma; Betsi Cadwaladr CHC, Paediatric Department<br>Patil, Manasi; Liverpool School of Tropical Medicine<br>Dickinson, Fiona; Liverpool School of Tropical Medicine<br>Godia, Pamela; Liverpool School of Tropical Medicine<br>Maua, Judith; Liverpool School of Tropical Medicine<br>Sammy, Mercy; Gertrude's Garden Children's Hospital<br>Naimoi, Bridget; AMPATH Kenya<br>Warfa, Osman; Kenya Ministry of Health<br>Dewez, Juan ; London School of Hygiene and Tropical Medicine Faculty of Epidemiology and Public Health<br>Murila, Florence; University of Nairobi School of Medicine<br>Manu, Alexander; Liverpool School of Tropical Medicine<br>Smith, Helen; International Health Consulting Services Ltd<br>Mathai, Matthews; Liverpool School of Tropical Medicine, Centre for Maternal and Newborn Health
**Primary Subject Heading**:	Paediatrics
Secondary Subject Heading:	Qualitative research
Keywords:	NEONATOLOGY, QUALITATIVE RESEARCH, RESPIRATORY MEDICINE (see Thoracic Medicine)

SCHOLARONE™
Manuscripts

I, the Submitting Author has the right to grant and does grant on behalf of all authors of the Work (as defined in the below author licence), an exclusive licence and/or a non-exclusive licence for contributions from authors who are: i) UK Crown employees; ii) where BMJ has agreed a CC-BY licence shall apply, and/or iii) in accordance with the terms applicable for US Federal Government officers or employees acting as part of their official duties; on a worldwide, perpetual, irrevocable, royalty-free basis to BMJ Publishing Group Ltd ("BMJ") its licensees and where the relevant Journal is co-owned by BMJ to the co-owners of the Journal, to publish the Work in this journal and any other BMJ products and to exploit all rights, as set out in our licence.

The Submitting Author accepts and understands that any supply made under these terms is made by BMJ to the Submitting Author unless you are acting as an employee on behalf of your employer or a postgraduate student of an affiliated institution which is paying any applicable article publishing charge ("APC") for Open Access articles. Where the Submitting Author wishes to make the Work available on an Open Access basis (and intends to pay the relevant APC), the terms of reuse of such Open Access shall be governed by a Creative Commons licence – details of these licences and which Creative Commons licence will apply to this Work are set out in our licence referred to above.

Other than as permitted in any relevant BMJ Author's Self Archiving Policies, I confirm this Work has not been accepted for publication elsewhere, is not being considered for publication elsewhere and does not duplicate material already published. I confirm all authors consent to publication of this Work and authorise the granting of this licence.

For peer review only

"Sometimes you are forced to play God…": a qualitative study of healthcare worker experiences of using continuous positive airway pressure in newborn care in Kenya

Helen M Nabwera,[1] Jemma L Wright[2], Manasi Patil[1], Fiona Dickinson[1], Pamela Godia[3], Judith Maua[3], Mercy K. Sammy[4], Bridget C. Naimoi[5], Osman H Warfa[6], Juan E Dewez[7], Florence Murila[8], Alexander Manu[1], Helen Smith[9], Matthews Mathai[1]

1. Centre for Maternal and Newborn Health, Liverpool School of Tropical Medicine, Pembroke Place, Liverpool L3 5QA, United Kingdom
2. Betsi Cadwaladr University Health Board, Wrexham Maelor Hospital, Croesnewydd Road, Wrexham, LL13 7TD
3. Liverpool School of Tropical Medicine, Wilson Business Park, Nairobi, Kenya
4. Gertrude's Children's Hospital, 34 Muthaiga Rd, Nairobi, Kenya
5. AMPATH, Eldoret, Kenya
6. Ministry of Health, Neonatal, Child and Adolescent Health Unit, Afya House, Cathedral Road, P.O. Box 30016–00100, Nairobi
7. London School of Hygiene and Tropical Medicine, Keppel street, London, WC1E 7HT, United Kingdom
8. University of Nairobi, Department of Paediatrics and Child Health & Kenyatta National Hospital, Kenya.
9. Independent consultant, International Health Consulting Services Ltd, Liverpool, United Kingdom.

Corresponding author:

Dr Helen M Nabwera

Liverpool School of Tropical Medicine, Pembroke Place,

Liverpool L3 5QA, United Kingdom

Phone: +44 (0) 151 702 9364

E-mail: helen.nabwera@lstmed.ac.uk

For peer review only

Abstract

Objective: To explore the experiences of using Continuous Positive Airway Pressure (CPAP) in newborn care among healthcare workers in Kenya, and to identify factors that would promote successful scale-up.

Design and setting: A qualitative study using key informant interviews and focus group discussions, based at secondary and tertiary level hospitals in Kenya.

Participants: Healthcare workers in the newborn units providing CPAP.

Primary and secondary outcome measure: Facilitators and barriers of CPAP use in newborn care in Kenya.

Results: 16 key informant interviews and 15 focus group discussions were conducted across 19 hospitals from September 2017 to February 2018. Main barriers reported were: 1) inadequate infrastructure to support the effective delivery of CPAP, 2) shortage of skilled staff rendering it difficult for the available staff to initiate or monitor infants on CPAP, 3) inadequate knowledge and training of staff that inhibited the safe care of infants on CPAP. Key facilitators reported were positive patient outcomes after CPAP use that increased staff confidence and partnership with caregivers in the management of newborns on CPAP. Healthcare workers in private/mission hospitals had more positive experiences of using CPAP in newborn care as the relevant support and infrastructure were available.

Conclusion: CPAP use in newborn care is valued by healthcare workers in Kenya. However, we identified key challenges that threaten its safe use and sustainability.

For peer review only - http://bmjopen.bmj.com/site/about/guidelines.xhtml

Further scale up of CPAP in newborn care should ensure that staff members have ready access to optimal training on CPAP and that there are enough resources and infrastructure to support its use.

Ethics: This study was approved through the appropriate ethics committees in Kenya and the United Kingdom (see in text) with written informed consent for each participant.

Strengths and limitations of this study

- First study in Kenya that has explored the experiences of healthcare workers using CPAP in newborn care in-depth, obtaining data from multiple departments with a diverse range of available services and different levels of access to resources.
- The study was timely as it was conducted approximately 2 years after an intense implementation programme of CPAP in Newborn Units in public hospitals, therefore providing key information on the sustainability of this exercise.
- The study was conducted at a time when there were several healthcare workers strikes that significantly disrupted health care services in Kenya, therefore the health care worker perspectives in this study may reflect the views of the subset who returned to work after the strikes.

For peer review only - http://bmjopen.bmj.com/site/about/guidelines.xhtml

Background

The neonatal period (i.e. first 28 days after birth) is well recognized globally as the most vulnerable time of childhood [1]. An estimated 2.5 million neonatal deaths occurred worldwide in 2017 accounting for 46% of all under 5 deaths [2]. The majority of these neonatal deaths occurred in low and middle-income countries (LMICs), with the highest burden in South Asia and sub-Saharan Africa (including Kenya) [2, 3].

Severe respiratory distress is a serious complication of the three leading causes of neonatal death (i.e. preterm birth, intra-partum related events and sepsis) with a case fatality rate of up to 20% in LMICs [4, 5]. The provision of respiratory support is therefore a key requirement for the care of hospitalised small and sick newborns, but is often inaccessible in many LMICs [4, 6].

Continuous Positive Airway Pressure (CPAP) is a relatively simple and cheap intervention that saves lives, particularly among preterm or very low birth weight neonates (weight<1,500g) with respiratory distress syndrome [6-10]. CPAP has been used in newborn care in high-income countries (HICs) for over 60 years [7]. The use of CPAP and oxygen therapy in HICs was associated with a 75% reduction in neonatal deaths due to respiratory distress syndrome (RDS) [7]. In a trial in Bangladesh, when compared to low-flow oxygen therapy alone, bubble CPAP reduced deaths in children under 5 with severe pneumonia and hypoxaemia by 75% [11]. In another trial in Malawi, CPAP reduced deaths by 27% in neonates weighing >1000g with

For peer review only

severe respiratory distress [12]. However, CPAP use is also associated with adverse events, such as nasal trauma, which are rarely reported in LMICs possibly due to staff limitations in being able to identify them [13-15].

Kenya is one of the 81 countries that account for 90% of child deaths worldwide [16]. Neonatal conditions including severe neonatal infections, birth asphyxia, preterm births and congenital anomalies are the leading cause of under 5 mortality [17]. The neonatal mortality rate in Kenya is higher than the overall global estimate (22 per 1000 live births in 2015) and has reduced slowly over the past few decades [17]. To address the deficiencies in neonatal care, CPAP use in newborn units in the public sector has been scaled up since 2014 [18-20]. In addition, the Ministry of Health in Kenya recommended the use of CPAP in newborn care in secondary and tertiary level hospitals and developed a national guideline to support its use in 2016 [21]. However, factors that influence the sustained and effective use of CPAP in newborn care, relevant to the Kenyan context, are largely unknown.

The aim of our study was therefore to explore the experiences of using CPAP in newborn care among healthcare workers, and to identify the barriers and facilitators of its use, thus informing the future scale-up of this life-saving intervention in Kenya.

Methods

Ethics approval

Written informed consent was obtained from each participant prior to enrolment.

For peer review only

For peer review only - http://bmjopen.bmj.com/site/about/guidelines.xhtml

Study Design

This was a mixed methods study in which we collected both qualitative data and quantitative data from the cross-sectional survey. In this paper, we present the results of the qualitative component of the study in which we conducted key informant interviews and focus group discussions among health care workers. This enabled us to gain an in-depth understanding of the gaps in providing CPAP in newborn care and to identify solutions in the context of a devolved, but resource-constrained public healthcare system, with different governance structures [22-26]. The inclusion of private/mission hospitals also enabled us to gain diverse perspectives on CPAP use in Kenya in the context of differing levels of resources.

Study Population and Setting

The study population included all healthcare workers involved in providing inpatient secondary or tertiary level newborn care services in Kenyan hospitals. Identification of these hospitals was based on information provided by the Division of Child and Adolescent Health at the Ministry of Health of Kenya and non-governmental organisations (NGOs) involved in newborn care in Kenya. Twenty-three hospitals provided CPAP in newborn care (18 public, 3 private "for-profit" and 2 mission "not-for-profit"). The study was conducted across 19 hospitals that were spread across 15 of the 47 counties of Kenya. These included 15 public referral hospitals (8 County, 6 Regional and 1 National level) and 2 private "for-profit" and 2 mission "not-for-profit" hospitals. These constituted 83% of all hospitals in Kenya that provided CPAP

For peer review only - http://bmjopen.bmj.com/site/about/guidelines.xhtml

newborn care. **(Table 1)**

Sampling

There were two phases of sampling in this study. Convenience sampling was used to recruit the 19 hospitals, which were the hospitals that gave us permission to access their facilities and staff to conduct our study [27]. Purposive sampling was used to recruit healthcare workers with any experience of working on the newborn units that offered CPAP. This enabled us to get a broad range of views and experiences on CPAP use within and between different newborn units in Kenya [28]. We estimated that at each hospital, a sample size of 10-12 participants would be sufficient to achieve data saturation, where no new themes would emerge from collecting more data from the site [29]. We included all hospitals that provided CPAP in their newborn units and were willing to participate in the study. Our sampling framework is outlined in **Table 2.**

The focus group discussions consisted of 6-7 healthcare workers with different levels of expertise in newborn care (including nursing students, nurses, medical officers, clinical officers and senior paediatricians), in order to get a perspective of the teamwork and decision-making process around CPAP use. For the key informant interviews, we interviewed senior nurses or doctors who we expected to have an appropriate level of knowledge of the operational aspects of CPAP use in their respective newborn units. As we conducted this work at a time when there were multiple health care worker strikes in Kenya, many newborn units were already short

For peer review only - http://bmjopen.bmj.com/site/about/guidelines.xhtml

staffed. However, we worked closely with the Ministry of Health, county governments and hospitals to ensure that we only collected our data on days when the units reported having adequate numbers of staff and could function for 1-2 hours with fewer staff in order to attend our interviews. On many occasions we had to cancel planned visits and wait for a more appropriate time.

Data Collection

Data was collected from September 2017 to February 2018. The key informant interview and focus group discussion guides focused on the following areas in the context of CPAP use in newborn care: training, decision-making for initiating or stopping, resources and interpersonal relationships. The guides were pilot tested with nurses at one of the county referral hospitals, and this process and data were used to refine the guides. Informed consent was sought from each participant prior to conducting the focus group discussions and key informant interviews. All interviews/discussions were conducted face-to-face at the respective hospitals by six research assistants (five female and one male). All research assistants were current or former practicing clinicians, all of whom had either used CPAP or were trained in its use. All research assistants had received 3-days of intensive training on conducting qualitative interviews/focus group discussions and were fluent in both English and Kiswahili. All interviews were predominately conducted in English, although some participants used terms in Kiswahili to explain their points. The interviews/focus group discussions were recorded and transcribed verbatim by two

For peer review only - http://bmjopen.bmj.com/site/about/guidelines.xhtml

of the research assistants and an independent transcriber. Each of the research assistants reviewed the transcripts for accuracy and discussed these with the lead investigator during debriefing sessions to establish the validity of the transcripts [30].

Data Analysis

We used thematic analysis [26, 31, 32]. Data analysis was an iterative process that started alongside data collection, allowing exploration of key emerging themes [32]. A conceptual framework was used to guide our analysis (**Figure 1**). After extensive familiarization with the data, 5 of the investigators (HN, MP, JW, MKS, BCN) developed a coding scheme, which was revised further during the analysis to ensure the robustness of the approach [33, 34]. NVivo 11 software (QSR International Pty Ltd 2015) was used for data management.

Patient and public involvement statement

This was a countrywide qualitative study that was conducted among healthcare workers working on newborn units in hospitals in Kenya in 2017/2018. This study was designed to explore healthcare workers experiences of using CPAP in newborn care in Kenya. No patients or members of the public were involved in the design, recruitment or conduct of the study. The results were disseminated to participants through the leadership in their departments and counties, after the study was completed.

For peer review only

Results

We conducted 16 key informant interviews and 15 focus group discussions among 100 healthcare workers in Kenya. The majority were nurses (56%) in the age range 31-40 years, and 77% were female. The overall median number of years of working in healthcare was 15 years (IQR 8, 17) and in newborn care 4 years (IQR 3, 5). **(Table 3)**

Although healthcare workers felt that the use of CPAP had the potential to enhance newborn care in Kenya, there were multiple challenges with implementation predominantly in the public sector. The key barriers were provision of inadequate infrastructure for effective delivery of CPAP; shortages of skilled staff that hindered CPAP initiation and management; and inadequate knowledge and training of CPAP among staff limiting its safe use. However, these barriers were sometimes mitigated by positive patient outcomes that increased staff confidence in CPAP use and promoted partnership between caregivers (parents or other key family members) and healthcare workers when initiating CPAP. Healthcare workers in better-resourced private/mission hospitals had more positive experiences of using CPAP in newborn care.

<u>Effect of inadequate infrastructure on optimal delivery of CPAP</u>

Overall, the infrastructure to support CPAP use in newborn care within the public sector was reported as inadequate. Healthcare workers highlighted that there were often not enough CPAP machines to meet patient demands, meaning that they

For peer review only

For peer review only - http://bmjopen.bmj.com/site/about/guidelines.xhtml

frequently faced the difficult decision of rationing CPAP.

"Sometimes you are forced to play God, which we don't like. We would want to have as many CPAP machines as possible but, yeah, it's not possible. So, this is the matter of weighing and looking at who is most likely to survive." (key informant interview, newborn unit 5, paediatrician, public hospital)

Staff described the ethical dilemma of discontinuing CPAP support inappropriately early in a baby that was improving in order to initiate it on a sicker baby. This was extremely distressing for staff.

"It is a psychological torture to us and even to them, to the mother. So, such experiences we have had them severally and even for us we feel guilty "If I could have done this for the baby they could have survived as these ones", you see" (focus group discussion, newborn unit 8, nurse, public hospital)

There were also frequent shortages of key CPAP consumables in newborn units, such as appropriately sized nasal prongs, due to difficulties with procurement. Healthcare workers reported that after the completion of donor-supported implementation, hospitals struggled to find affordable suppliers for these consumables. The newborn units therefore either improvised or reused equipment that was supposed to be disposed of, posing an infection risk to the newborn and increasing the risk of other adverse events.

" Like the nasal prongs size zero is usually a challenge…sometimes they go out of

For peer review only - http://bmjopen.bmj.com/site/about/guidelines.xhtml

stock…we usually wash them, they are actually supposed to be disposed but we are allowed to wash them even if they are not visibly dirty, but even if you washed them, it reaches that time you must throw them." (focus group discussion, newborn unit 8, nurse, public hospital)

General infrastructure difficulties also meant that use of CPAP was restricted by other issues, such as unreliable power and oxygen supplies, and lack of physical space in the newborn units for the CPAP machinery.

"At times our electricity is on and off, I remember a time a baby was on CPAP and our generator had a problem and we were told to wait for around thirty-five minutes for them to fuel the generator. So, it interfered with that process." (focus group discussion, newborn unit 14, nurse, public hospital)

"Another factor is the availability of space, like in our nursery when you come when we have babies, we just put babies close, …and CPAP needs a spacious place. So sometimes you can have a challenge starting it, because where will you put these babies so that you make a space for that child." (focus group discussion, newborn unit 8, nurse, public hospital)

The data also highlighted a lack of other relevant resources required to treat neonates, specifically neonatal medications and laboratory support.

"Again, the other challenge is the laboratory investigations, the baby is on CPAP, maybe you will need investigation like blood cultures, but you find that the hospital

For peer review only

does not have enough reagents… So, this is a baby on CPAP but you cannot even investigate" (focus group discussion, newborn unit 9, nurse, public hospital)

Shortages of skilled staff that hindered CPAP initiation and management

Staffing issues across most newborn units were identified as a key barrier to the use of CPAP, either due to staff shortages, a high staff turnover or healthcare worker strikes. It was highlighted that babies on CPAP need extra monitoring by the nursing staff, so staff shortages were felt to directly limit CPAP use on the newborn units. Experiences included times on busy shifts when the staff did not put babies on CPAP, using oxygen instead due to the extra monitoring required for babies on CPAP, even though they knew this would result in suboptimal care for the baby.

"You find you are only two with more than twenty babies in the NBU, you feel if I fix (CPAP), I am still doing other things and I will not be able to observe the baby as required" (focus group discussion, newborn unit 6, nurse, public hospital)

Unfortunately, there were several prolonged healthcare providers' strikes in the public sector during our data collection, which disrupted CPAP use. These strikes resulted in reduced confidence in using CPAP amongst healthcare providers, many of who had received in-house training but were dependent on resources or senior support lost during the strikes.

"We have the cards (containing instructions for setting up CPAP)…With the strike right now I don't know exactly where they are but I think we are going to find them

For peer review only - http://bmjopen.bmj.com/site/about/guidelines.xhtml

somehow." (focus group discussion, newborn unit 15, nurse, public hospital)

Inadequate knowledge and training for appropriate CPAP use

The introduction of CPAP to newborn units in the public sector often occurred with support of external NGOs. The acquisition of CPAP machines was accompanied by initial training of the senior members of staff in each unit, with a plan to cascade this training to other staff members during in-house educational meetings.

"I think one of the things that has made it work in this hospital is the good training that has been passed on from the pioneer group that was trained." (focus group discussion, newborn unit 17, medical officer, public hospital)

However inadequate training of staff was reported in most of the newborn units as the plan to cascade the training often did not occur. In some cases, the most senior members of the team with skills for using CPAP left and were replaced with new staff members untrained in the use of CPAP. This resulted in a decline in the skills and confidence of using CPAP among healthcare workers in these units to the point that CPAP was no longer used even though the equipment was available and in working order.

"Just after the training, I think our paediatrician left the facility and from that time, utilization and prescription of CPAP was very, very minimal and kept deteriorating until now it is like nobody is thinking about CPAP" (focus group discussion, newborn unit 6, senior nurse, public hospital)

For peer review only - http://bmjopen.bmj.com/site/about/guidelines.xhtml

On-going training on CPAP was variable with staff from private/mission hospitals describing regular and structured training, whilst most public sector healthcare workers only received informal on-the-job training. This training was very dependent on the level of support for CPAP within the unit and the presence of key educators. From our data we found that training occurred more regularly in private and mission hospitals, where there was access to better resources and fewer admissions.

"Once they get the training, they are very positive and also willing to train others. So, it does not matter whoever is training, so long as you have been trained by somebody else it is something you pass to the next person." (focus group discussion, newborn unit 17, senior nurse, public hospital)

However, even with regular training, the frequent rotation of staff between hospital departments was also noted to have a significant impact on the competencies of the staff.

"One of the biggest challenge is the structure of the healthcare system because the people we started with, the ones who were trained were changed, and a new team comes which is totally not competent and you tell them about CPAP and they fear." (key informant interview, newborn unit 9, public hospital)

Inadequate numbers of staff with skills for using CPAP also hindered staff from initiating this therapy, and healthcare workers reported that many of them had not received training on using CPAP. As a result, some reported witnessing inappropriate

For peer review only

cessation of CPAP when a patient was handed over to another member of staff.

"So, if I'm working in the morning, I would be able to use CPAP. There is the other person who comes by night. If they feel they are not comfortable in the use of CPAP, they wouldn't put children on CPAP." (key informant interview, newborn unit 5, public hospital)

The healthcare workers also had limited knowledge about the identification and management of common CPAP complications, resulting in potentially unsafe use of CPAP.

"I will not deny because I have not been taught how to identify complications on CPAP." (focus group discussion, newborn unit 14, medical officer, public hospital)

Notably, the presence of a 'CPAP champion' in a newborn unit was noted to improve the frequency of training and level of knowledge about CPAP among the staff members. Good leadership was critical in ensuring that all staff members were trained to use CPAP.

"We have 2 champion nurses, we have the nursing officer in charge…she got to understand how they connect, and what are the essential things with the CPAP, then now what she does is she trains any new nurses who come through." (key informant interview, newborn unit 12, public hospital)

Positive infant outcomes that increased staff confidence in CPAP use

For peer review only - http://bmjopen.bmj.com/site/about/guidelines.xhtml

Staff in NBUs felt that CPAP was useful in improving neonatal care when used appropriately. Indeed, personal experience of good outcomes associated with CPAP use was identified as improving staff morale and their confidence in using CPAP.

"There are some babies we had managed there who had (a) bad prognosis and sometimes you put (them) on CPAP and you see the baby is not in (a) very good state. As I have said the prognosis is bad, but you put on CPAP and you monitor, and they pull through. Even other healthcare providers, they get surprised" (focus group discussion, newborn unit 14, medical officer, public hospital)

However, there was recognition that when it was used inappropriately, for example when the recommended criteria for initiating or stopping CPAP were not adhered to, the outcomes were poor. This resulted in diminished confidence of CPAP use among staff and had a negative impact on its sustainability in those units.

"It has instilled fear among the nurses such that you take them through the training but when you tell them to go and start, you find that they fear because they don't want to be associated with starting a CPAP and a baby dying, that is now the problem." (focus group discussion, newborn unit 9, nurse, public hospital)

High staff confidence and engagement was identified as important in promoting the use of CPAP. The empowerment of all members of the clinical team to initiate CPAP on newborns who meet the criteria was important in promoting its use, particularly in reducing delays in starting this intervention. This was especially noted if staff members were well supported by the leadership of the hospital.

For peer review only

For peer review only - http://bmjopen.bmj.com/site/about/guidelines.xhtml

"The CPAP is good because the nurse can assess prescribe and administer even without a doctor and when the outcome is so good, it's motivating to the nurses. When you work like that you get so excited you feel you're empowered, and you feel like doing it more and more times." (focus group discussion, newborn unit 8, nurse, public hospital)

Effect of partnerships between caregivers and healthcare workers on uptake of CPAP

Use of appropriate communication skills that involved the caregivers in the decision-making of care strategies for their infants was felt to enhance acceptability of CPAP.

"Our policy is that they really have to consent, and not just consent in feeling that you are coercing them...Our target is to end up with a win-win, have a mutual agreement. It makes our work easier, and makes the baby get well faster. Because we are working as a team" (focus group discussion, newborn unit 1, nurse, private hospital)

Caregivers were also reported to be more receptive to the use of CPAP if they were actively encouraged to be involved in the care of their baby whilst on CPAP.

'The mother or the caregiver must be aware, you must counsel them, they accept before you start CPAP because if they refuse then it means they'll interfere with the technical bit of it." (focus group discussion, newborn unit 8, nurse, public hospital)

Overall, in units where the outcomes of the babies on CPAP were good, the

For peer review only

healthcare workers reported that both the main caregivers and the wider community became more receptive to CPAP.

“It’s a bit tricky for ... if you put CPAP and a child passes on, nobody will now accept CPAP, no one will accept. If the one improves, the next one also wants it”. (focus group discussion, newborn unit 5, nurse, public hospital)

Discussion

Our study describes experiences of healthcare workers using CPAP within newborn units in Kenya. The implementation gaps were predominantly in the public sector and these were mainly at the health system level. However, we also found factors at a personal level for both caregivers and healthcare providers that facilitated CPAP use, even in the context of resource limitations.

Our study found that CPAP was felt to be beneficial when the right training and infrastructure was in place for its use. This is consistent with findings from an observational study in a rural Ugandan neonatal intensive care unit (NICU), which found that training using the Silverman-Anderson respiratory severity score made it feasible to implement CPAP in newborn care safely in a resource-limited setting [35]. Another study in a rural mission hospital in Kenya showed higher survival-to-discharge rates in premature infants with severe respiratory distress syndrome after the introduction of CPAP with adequate staff training and infrastructure to support its use [19]. In Malawi, a non-randomized controlled study also showed a 27% absolute improvement in survival of neonates with severe RDS [12] and that CPAP

For peer review only - http://bmjopen.bmj.com/site/about/guidelines.xhtml

was a cost-effective strategy for the care of newborns with respiratory difficulty when compared to nasal oxygen therapy [36]. Our data suggests that poor infrastructure is a major hindrance to the safe and effective use of CPAP in Kenyan public hospitals, especially once external funding to support CPAP implementation including provision of adequate equipment, staff training and tools to monitor it safe use, ends.

The infrastructure and supplies for CPAP use were inadequate particularly in the public sector where sustaining CPAP use after the completion of donor funded programmes was hampered by the challenges with procurement of consumables. As a result, newborns were potentially being exposed to risks such as infections (due to prolonged use or recycling of respiratory circuits), hence limiting its benefit to these vulnerable babies. In addition, staff had to frequently ration CPAP, which sometimes meant deviation from the national guidelines for stopping CPAP in order to try and meet the demand. In a related study in India, the frustration of the lack of resources to deliver CPAP optimally in newborn care units left staff feeling despondent and demotivated, which had implications for the scale up of this intervention [37]. Indeed, our data from Kenya showed that when staff were not able to deliver CPAP optimally and had newborn mortalities when implementing it, this resulted in fear and anxiety of using CPAP. This fear of CPAP ultimately resulted in decreased use therefore hindering the global strategies to end preventable deaths in newborns [38].

For peer review only - http://bmjopen.bmj.com/site/about/guidelines.xhtml

The inadequate number of skilled staff, particularly nursing staff, was a major challenge. Nurses within our study reported being reluctant to start CPAP use due to poor nurse: patient ratios leading to the inability to monitor these babies adequately while on CPAP - a WHO prerequisite for using CPAP [8, 39]. Inadequate staffing of newborn units is a major challenge in Kenya, with the current median nurse: baby ratio in the public sector reported to be as high as 17 babies per nurse [40]. Recent exploratory work in hospitals in Nairobi, Kenya found that nurses had developed strategies to cope with their workload by task-shifting, whereby less technical tasks are shifted to student nurses, other support staff or mothers to free-up nursing time to focus on the more technical aspects of newborn care [41]. Future projects are planned that will seek to formalise this task-shifting to support nurses working in these busy newborn units, which may also have an impact on the delivery of CPAP [41].

Inadequate training of healthcare workers was another common problem identified within our data that had a negative impact on CPAP use. A recent cross-sectional study from Kenya also found that over a third of sick newborns were cared for in an environment where nursing knowledge was very low [42]. When CPAP was introduced in public hospitals in western Kenya, a successful training-of-trainers model was used [18], but our findings suggest that its effectiveness was not sustained over time. Regular staff rotations and prolonged healthcare worker strikes in the public sector appears to have had a negative impact on CPAP related competencies of staff. In the context of limited training opportunities, work has been

For peer review only

For peer review only - http://bmjopen.bmj.com/site/about/guidelines.xhtml

done in Malawi to develop a simple clinical algorithm, to identify newborns who would benefit most from CPAP, with evidence of high inter-rater reliability, sensitivity and specificity for their new 'TRY' tool [43, 44]. In addition, new training-of-trainers programs [45] and health partnership models, where international experts support training strategies over a longer period of time [46], could be beneficial within Kenya. Sustained refresher training on the use of CPAP in newborn care units in LMICs is also crucial. Indeed, a recent retrospective cohort study in rural Rwanda that found that even after training staff, identification of eligible newborns was a challenge [47].

Finally, our findings also suggest that partnership between healthcare workers and the main caregivers in the decision-making surrounding CPAP use in their babies is critical to the acceptability of this intervention. Internationally, there is a growing recognition of the importance of involving parents in the care of their hospitalized newborns in improving outcomes for both the infant and their family [48]. A descriptive study conducted at a tertiary newborn unit in Malawi found that caregivers felt anxious and fearful when their infants were on CPAP with limited parent-child interaction [49]. However, this anxiety was mitigated by support from healthcare providers, family and friends [49]. In Rwanda a 'parental neonatal curriculum' has recently been developed using a Delphi consensus technique involving parents and healthcare workers [50]. The development of such curricula has the potential to enhance true partnership with parents [50]. In Malawi, Family-Led Care Models have recently been implemented to improve the facility and home-

For peer review only - http://bmjopen.bmj.com/site/about/guidelines.xhtml

based care of preterm/low birth weight newborns [51]. The evaluation of the impact of these strategies is keenly awaited.

The key limitation of this study was that it was conducted at a time when there were several healthcare workers strikes that significantly disrupted health care services in Kenya. Our findings may therefore reflect the views predominantly of healthcare workers who returned to work after the strikes. As a result, our data predominantly contains the perspectives of nurses, who were the frontline staff involved in delivering CPAP in newborn care in Kenyan hospitals. Unfortunately, we were unable to compare the differing perspectives of doctors and nurses in order to highlight any differences in staff experience in line with their professional background. It would have been useful to interview doctors and nurses independently in order to identifying any potential team working challenges that might have influenced decision making and the delivery of CPAP in newborn care in the Kenyan health system.

Another limitation of this study is the diversity of healthcare workers present in our focus group discussions, which may have led to reduced reporting of the experiences of the junior members of the healthcare team. Holding such heterogeneous focus group discussions might have meant that the more junior colleagues felt less able to express their opinions due to the presence of their more senior colleagues in a hierarchical health care system. Ideally more homogeneous focus groups with regard to seniority or level of education or experience would have been better for more

For peer review only

For peer review only - http://bmjopen.bmj.com/site/about/guidelines.xhtml

enhanced interactions [52]. However, it was not possible because holding a focus group discussion with junior staff and then a separate one with senior staff due to staff shortages during the period of data collection and would have kept essential staff away from clinical duties for longer.

Finally, we did not include the perspectives of the parents/cargeivers or their families, a key group of stakeholders when evaluating an intervention within a health care system. Their perspectives would have given us a better understanding of the acceptability of CPAP in newborn care in Kenya and the effect of partnership between caregivers and healthcare workers on uptake of CPAP.

Nevertheless, to our knowledge, this is the first study in Kenya that has explored in-depth the experiences of healthcare workers using CPAP in newborn care. Our study involved healthcare workers in both the public and private/mission sectors of Kenya, enabling us to identify a diverse range of perspectives from healthcare professionals within the same country but with access to different levels of resources for providing newborn care. These findings will be pertinent for the future scale up strategies for CPAP and other newborn care technologies in Kenya and other similar settings.

Conclusion

The scale-up of CPAP use in newborn care in Kenya is a strategy that healthcare workers value as a key intervention for improving the survival of newborns. However, there are important challenges that need to be addressed to ensure sustainability and safety of its use in Kenyan hospitals. Worryingly, newborns are

For peer review only

For peer review only - http://bmjopen.bmj.com/site/about/guidelines.xhtml

currently exposed to preventable risks, limiting the benefit of CPAP in these vulnerable babies. Future CPAP implementation strategies should ensure that infrastructure for CPAP use is fit for purpose, shortages of skilled staff are addressed, and that staff training and partnership with caregivers are prioritised.

Acknowledgements

We would like to thank the health care workers at the Newborn Units of the following hospitals: Aga Khan University Hospital, Nairobi; Bungoma County Referral Hospital; Busia County Referral Hospital; Coast General Hospital, Mombasa; Gertrude's Children's Hospital, Nairobi; Homabay County Referral Hospital; Jaramogi Oginga Odinga Teaching and Referral Hospital, Kisumu; Kakamega County Referral Hospital; Kenyatta National Hospital; Kiambu County Hospital; Kijabe Mission Hospital; Kisii County Referral Hospital; Kisumu County Referral Hospital; Machakos County Referral Hospital; Mama Lucy Hospital, Nairobi; Migori County Referral Hospital; Naivasha Sub-county Hospital; Pumwani Maternity Hospital, Nairobi; Siaya County Referral Hospital; Tenwek Mission Hospital, Bomet. We are grateful also to country health representatives and the Ministry of Health in Kenya for their participation and support in conducting this study. Many thanks to our colleagues Ms Joyce Mutuku, Mr Onesimus Muchemi, Ms Sylvia Gichuru, Ms Linda Omolo, Ms Beatrice Ochieng who supported us with data collection. We thank Dr Sarah White, Ms Sophie Ngugi and Mr Allan Govoga for their important contributions to the conceptualization of this study. We thank Dr Bernard Olayo and Miss Caroline Kendi

For peer review only - http://bmjopen.bmj.com/site/about/guidelines.xhtml

(Centre for Public Health and Development, Kenya) for their advice and support.

Contributors

HMN coordinated the study, analyzed the data, prepared the manuscript and approved the final manuscript as submitted. She took over as Principal Investigator of the study prior to the commencement of data collection and coordinated all aspects of the project thereafter. JW, MP analyzed the data and prepared the manuscript and approved the final manuscript as submitted. FD led the data management of this study, reviewed and approved the final manuscript as submitted. She was the UK-based Research Assistant in this study. PG conceptualised the study, reviewed and approved the final manuscript as submitted. She led with the coordination of the ethics approval processes in Kenya. JM conceptualised the study, provided support with the coordination of study activities, reviewed and approved the final manuscript as submitted. She led the communication strategies with the Kenyan stakeholders. MKS and BCN collected, transcribed and analyzed the data. They were Research Assistants in this study in Kenya. OHW conceptualised the study, reviewed and approved the final manuscript as submitted. He facilitated the approval of study activities at the national and facility level. JED conceptualised the study, reviewed and approved the final manuscript as submitted. He was the initial Principal Investigator of the study and led the ethics application process. FM conceptualised the study, reviewed and approved the final manuscript as submitted. She was co-Principal Investigator and supported the ethics application processes of

For peer review only - http://bmjopen.bmj.com/site/about/guidelines.xhtml

the study in Kenya. She also provided very valuable context-relevant information during the data collection and analysis phases. AM supported with study coordination, reviewed and approved the final manuscript as submitted. HS provided guidance on the qualitative methodology during the conceptualization of the study, reviewed and approved the and approved the final manuscript as submitted. She was the lead Social Scientist for this study and provided critical review of the data collection and analysis. MM conceptualised the study, reviewed and approved the and approved the final manuscript as submitted. He provided oversight for all the study processes.

Funding

This project was funded by the UK Department for International Development (Project number: 202549), awarded to Prof Nynke van den Broek.

Competing interests

None declared.

Patient consent

Not required.

Ethics approval

The study was approved by the Research and Ethics Committee at the Liverpool School of Tropical Medicine (protocol number:15-032) and the University of Nairobi-

For peer review only

Kenyatta National Hospital Ethics Research Committee (P56/02/2017).

Provenance and peer review

Not commissioned; externally peer reviewed.

Data sharing statement

All data relevant to the study are included in the article. Extra data is available by emailing matthews.mathai@lstmed.ac.uk.

Open access

This is an open access article distributed in accordance with the Creative Commons Attribution Non Commercial (CC BY-NC 4.0) license, which permits others to distribute, remix, adapt, build upon this work non-commercially, and license their derivative works on different terms, provided the original work is properly cited, appropriate credit is given, any changes made indicated, and the use is non-commercial. See: http://creativecommons.org/licenses/by-nc/4.0/.

For peer review only - http://bmjopen.bmj.com/site/about/guidelines.xhtml

References

1. **Neonatal mortality** [https://data.unicef.org/topic/child-survival/neonatal-mortality/]
2. UN-IGME.: **Levels and Trenda in Child Mortality**. In. New York; 2018.
3. Lawn JE, Blencowe H, Oza S, You D, Lee AC, Waiswa P, Lalli M, Bhutta Z, Barros AJ, Christian P *et al*: **Every Newborn: progress, priorities, and potential beyond survival.** *Lancet* 2014, **384**(9938):189-205.
4. Duke T: **CPAP and high-flow oxygen to address high mortality of very severe pneumonia in low-income countries - keeping it in perspective**. *Paediatr Int Child Health* 2019, **39**(3):155-159.
5. UN-IGME.: **Levels and trends in child mortality: Report 2017**. In. New York, USA: UN-Interagency group for child mortality estimation; 2017.
6. Duke T: **CPAP: a guide for clinicians in developing countries**. *Paediatr Int Child Health* 2014, **34**(1):3-11.
7. Ho JJ, Subramaniam P, Davis PG: **Continuous distending pressure for respiratory distress in preterm infants**. *Cochrane Database Syst Rev* 2015(7):CD002271.
8. WHO: **WHO recommendation on continuous positive airway pressure therapy for the treatment of preterm newborns with respiratory distress syndrome**. In. Geneva: WHO; 2015.
9. Okello F, Egiru E, Ikiror J, Acom L, Loe K, Olupot-Olupot P, Burgoine K: **Reducing preterm mortality in eastern Uganda: the impact of introducing low-cost bubble CPAP on neonates <1500 g**. *BMC Pediatr* 2019, **19**(1):311.
10. Ezenwa B, Akintan P, Fajolu I, Ladele J, Ezeaka C: **Bubble CPAP in the Management of Respiratory Distress Syndrome in Resource Constrained Settings: The Luth Experience.** *Pediatr Oncall J* 2016, **13**:9-12.
11. Chisti MJ, Salam MA, Smith JH, Ahmed T, Pietroni MA, Shahunja KM, Shahid AS, Faruque AS, Ashraf H, Bardhan PK *et al*: **Bubble continuous positive airway pressure for children with severe pneumonia and hypoxaemia in Bangladesh: an open, randomised controlled trial**. *Lancet* 2015, **386**(9998):1057-1065.
12. Kawaza K, Machen HE, Brown J, Mwanza Z, Iniguez S, Gest A, Smith EO, Oden M, Richards-Kortum RR, Molyneux E: **Efficacy of a low-cost bubble CPAP system in treatment of respiratory distress in a neonatal ward in Malawi.** *PLoS One* 2014, **9**(1):e86327.
13. Lissauer T, Duke T, Mellor K, Molyneux L: **Nasal CPAP for neonatal respiratory support in low and middle-income countries**. *Arch Dis Child Fetal Neonatal Ed* 2017, **102**(3):F194-F196.
14. Dewez JE, van den Broek N: **Continuous positive airway pressure (CPAP) to treat respiratory distress in newborns in low- and middle-income countries**. *Trop Doct* 2017, **47**(1):19-22.
15. Fischer C, Bertelle V, Hohlfeld J, Forcada-Guex M, Stadelmann-Diaw C,

For peer review only - http://bmjopen.bmj.com/site/about/guidelines.xhtml

Tolsa JF: **Nasal trauma due to continuous positive airway pressure in neonates**. *Arch Dis Child Fetal Neonatal Ed* 2010, **95**(6):F447-451.

16. Countdown to C: **Countdown to 2030: tracking progress towards universal coverage for reproductive, maternal, newborn, and child health**. *Lancet* 2018, **391**(10129):1538-1548.
17. **Kenya: neonatal and child health profile** [https://www.who.int/pmnch/media/membernews/2011/20121216_kenyaparliament.pdf Accessed 12 June 2019]
18. Olayo B, Kirigia CK, Oliwa JN, Agai ON, Morris M, Benckert M, Adudans S, Murila F, Wilson PT: **Effective training-of-trainers model for the introduction of continuous positive airway pressure for neonatal and paediatric patients in Kenya**. *Paediatr Int Child Health* 2019, **39**(3):193-200.
19. Myhre J, Immaculate M, Okeyo B, Anand M, Omoding A, Myhre L, Okeyo L, Barasa I, Letchford S: **Effect of Treatment of Premature Infants with Respiratory Distress Using Low-cost Bubble CPAP in a Rural African Hospital**. *J Trop Pediatr* 2016, **62**(5):385-389.
20. Murphy GAV, Omondi GB, Gathara D, Abuya N, Mwachiro J, Kuria R, Tallam-Kimaiyo E, English M: **Expectations for nursing care in newborn units in Kenya: moving from implicit to explicit standards.** *BMJ Glob Health* 2018, **3**(2):e000645.
21. **Basic Paediatric Protocols** [https://www.psk.or.ke/public/uploads/file/c0d3675787d651dedbf4a0edfc9a2898.pdf Accessed 19 August 2019]
22. Pyone T, Smith H, van den Broek N: **Implementation of the free maternity services policy and its implications for health system governance in Kenya**. *BMJ Glob Health* 2017, **2**(4):e000249.
23. McCollum R, Limato R, Otiso L, Theobald S, Taegtmeyer M: **Health system governance following devolution: comparing experiences of decentralisation in Kenya and Indonesia**. *BMJ Glob Health* 2018, **3**(5):e000939.
24. McCollum R, Taegtmeyer M, Otiso L, Muturi N, Barasa E, Molyneux S, Martineau T, Theobald S: **"Sometimes it is difficult for us to stand up and change this": an analysis of power within priority-setting for health following devolution in Kenya**. *BMC Health Serv Res* 2018, **18**(1):906.
25. Al-Busaidi ZQ: **Qualitative research and its uses in health care**. *Sultan Qaboos Univ Med J* 2008, **8**(1):11-19.
26. Patton M: **Qualitative evaluation and research methods**. Beverly Hills, C.A.: Sage; 1990.
27. Gravetter FJ, Forzano LB: **Selecting Research Participants**. In: *Res Methods Behav Sci.* Sixth edn. U.S.A: Cengage; 2018.
28. Mays N, Pope C: **Qualitative research in health care. Assessing quality in qualitative research.** *BMJ* 2000, **320**(7226):50-52.
29. Guest G, Bunce A, Johnson L: **How Many Interviews Are Enough? An Experiment with Data Saturation and Variability**. *Field Methods* 2006,

For peer review only - http://bmjopen.bmj.com/site/about/guidelines.xhtml

18(1):59-82.
30. Kvale S: **An introduction to qualitative research**: SAGE; 1996.
31. Pope C, Ziebland S, Mays N: **Qualitative research in health care. Analysing qualitative data**. *BMJ* 2000, **320**(7227):114-116.
32. Braun V, Clarke V: **Using thematic analysis in psychology**. *Qualitative Research in Psychology* 2006, **3**(2):77-101.
33. Tuckett AG: **Applying thematic analysis theory to practice: A researcher's experience.** *Contemporary Nurse* 2005, **19**(1-2):75-87.
34. Carey JW, Morgan M, Oxtoby MJ: **Intercoder Agreement in Analysis of Responses to Open-Ended Interview Questions: Examples from Tuberculosis Research** *Cultural Anthropology Methods* 1996, **8**(3):1-5.
35. McAdams RM, Hedstrom AB, DiBlasi RM, Mant JE, Nyonyintono J, Otai CD, Lester DA, Batra M: **Implementation of Bubble CPAP in a Rural Ugandan Neonatal ICU**. *Respir Care* 2015, **60**(3):437-445.
36. Chen A, Deshmukh AA, Richards-Kortum R, Molyneux E, Kawaza K, Cantor SB: **Cost-effectiveness analysis of a low-cost bubble CPAP device in providing ventilatory support for neonates in Malawi - a preliminary report**. *BMC Pediatr* 2014, **14**:288.
37. Dewez JE, Chellani H, Nangia S, Metsis K, Smith H, Mathai M, van den Broek N: **Healthcare workers' views on the use of continuous positive airway pressure (CPAP) in neonates: a qualitative study in Andhra Pradesh, India.** *BMC Pediatr* 2018, **18**(1):347.
38. WHO: **Every newborn action plan**. In. Geneva, Switzerland: WHO; 2014.
39. WHO: **WHO recommendations on interventions to improve preterm birth outcomes.** In. Geneva: WHO; 2015.
40. Gathara D, Serem G, Murphy GAV, Obengo A, Tallam E, Jackson D, Brownie S, English M: **Missed nursing care in newborn units: a cross-sectional direct observational study**. *BMJ Qual Saf* 2019.
41. Nzinga J, McKnight J, Jepkosgei J, English M: **Exploring the space for task shifting to support nursing on neonatal wards in Kenyan public hospitals**. *Hum Resour Health* 2019, **17**(1):18.
42. Murphy GAV, Gathara D, Mwaniki A, Nabea G, Mwachiro J, Abuya N, English M: **Nursing knowledge of essential maternal and newborn care in a high-mortality urban African setting: A cross-sectional study**. *J Clin Nurs* 2019, **28**(5-6):882-893.
43. Hundalani SG, Richards-Kortum R, Oden M, Kawaza K, Gest A, Molyneux E: **Development and validation of a simple algorithm for initiation of CPAP in neonates with respiratory distress in Malawi.** *Arch Dis Child Fetal Neonatal Ed* 2015, **100**(4):F332-336.
44. Crehan C, Colbourn T, Heys M, Molyneux E: **Evaluation of 'TRY': an algorithm for neonatal continuous positive airways pressure in low-income settings**. *Arch Dis Child* 2018, **103**(8):732-738.
45. Wilson PT, Benckert MM, Moresky RT, Morris MC: **Development and Implementation of a Training-of-Trainers Program for Continuous Positive Airway Pressure in Neonatal and Pediatric Patients in Five Low- and Middle-Income Countries**. *J Trop Pediatr* 2017, **63**(5):358-

For peer review only - http://bmjopen.bmj.com/site/about/guidelines.xhtml

364.
46. Ntigurirwa P, Mellor K, Langer D, Evans M, Robertson E, Tuyisenge L, Groves A, Lissauer T: **A health partnership to reduce neonatal mortality in four hospitals in Rwanda**. *Global Health* 2017, **13**(1):28.
47. Nahimana E, Ngendahayo M, Magge H, Odhiambo J, Amoroso CL, Muhirwa E, Uwilingiyemungu JN, Nkikabahizi F, Habimana R, Hedt-Gauthier BL: **Bubble CPAP to support preterm infants in rural Rwanda: a retrospective cohort study**. *BMC Pediatr* 2015, **15**:135.
48. Banerjee J, Aloysius A, Platonos K, Deierl A: **Family centred care and family delivered care – What are we talking about?** *J of Neonatal Nursing* 2018, **24**(1):8-12.
49. Gondwe MJ, Gombachika B, Majamanda MD: **Experiences of caregivers of infants who have been on bubble continuous positive airway pressure at Queen Elizabeth Central Hospital, Malawi: A descriptive qualitative study**. *Malawi Med J* 2017, **29**(1):10-15.
50. Musabyemungu JA, Willson A, Batenhorst S, Webbe J, Cartledge PT: **What topics should we teach the parents of admitted neonates in the newborn care unit in the resource-limited setting - a Delphi study**. *Matern Health Neonatol Perinatol* 2019, **5**:11.
51. **Every Preemie scale** [https://www.everypreemie.org/malawi/family-led-care Accessed 13 August 2019]
52. McLafferty I: **Focus group interviews as a data collecting strategy**. *J Adv Nurs* 2004, **48**(2):187-194.

For peer review only - http://bmjopen.bmj.com/site/about/guidelines.xhtml

TABLES

Table 1: List of Newborn Baby Units with CPAP by Kenyan County

Name of County	Population of County (millions)	Number of Newborn Baby Units using CPAP
**Nairobi**	3.1	6
**Kiambu**	1.6	2
**Machakos**	1.1	1
**Nakuru**	1.6	2
**Mombasa**	0.9	1
**Uasin Gishu**	0.9	1
**Bomet**	0.7	1
**Kisumu**	0.3	2
**Homa Bay**	0.9	1
**Siaya**	0.8	1
**Migori**	0.9	1
**Kisii**	0.2	1
**Bungoma**	1.4	1
**Kakamega**	1.7	1
**Busia**	0.7	1
**Total**		23

Table 2: Sampling Framework

**Number of Hospitals using CPAP in newborn care**	23		
**Type of Hospital**	18 Public	3 Private or 2 Mission	
**Hospitals in Study**	15 Public	2 private	2 mission

For peer review only

For peer review only - http://bmjopen.bmj.com/site/about/guidelines.xhtml

Table 3: Characteristics of study participants

	National referral hospital (Level 6) N=8	Regional referral hospital (Level 5) N=36	County referral hospital (Level 4) N=36	Private N=8	Mission N=12	Overall N=100
**Majority age group, years**	31-40,<br><br>41-50	18-30	31-40	31-40	18-30	31-40
**Females, n (%)**	7 (88)	26 (72)	27 (77)	8 (100)	9 (75)	77 (77)
**Staff cadre**						
**Medical students, n**	1					1
**Nurses/midwives, n**	5	20	19	6	6	56
**Clinical officers, n**		4	1		1	6
**Medical officers, n**	3	9	11	1	3	27
**Paediatricians, n**		3	4	1	2	10
**Years of experience in health care, median (IQR)**	17 (9, 23)	15 (4, 23)	8 (2, 20)	17 (12, 19)	2 (2, 6)	15 (8, 17)
**Years of experience in newborn care, median (IQR)**	4 (3, 10)	5 (2, 10)	3 (1, 6)	5 (4, 7)	2 (1, 4)	4 (3, 5)

For peer review only

For peer review only - http://bmjopen.bmj.com/site/about/guidelines.xhtml

FIGURE LEGEND

Figure 1: Conceptual framework used to guide data analysis

For peer review only

Figure 1: Conceptual Framework

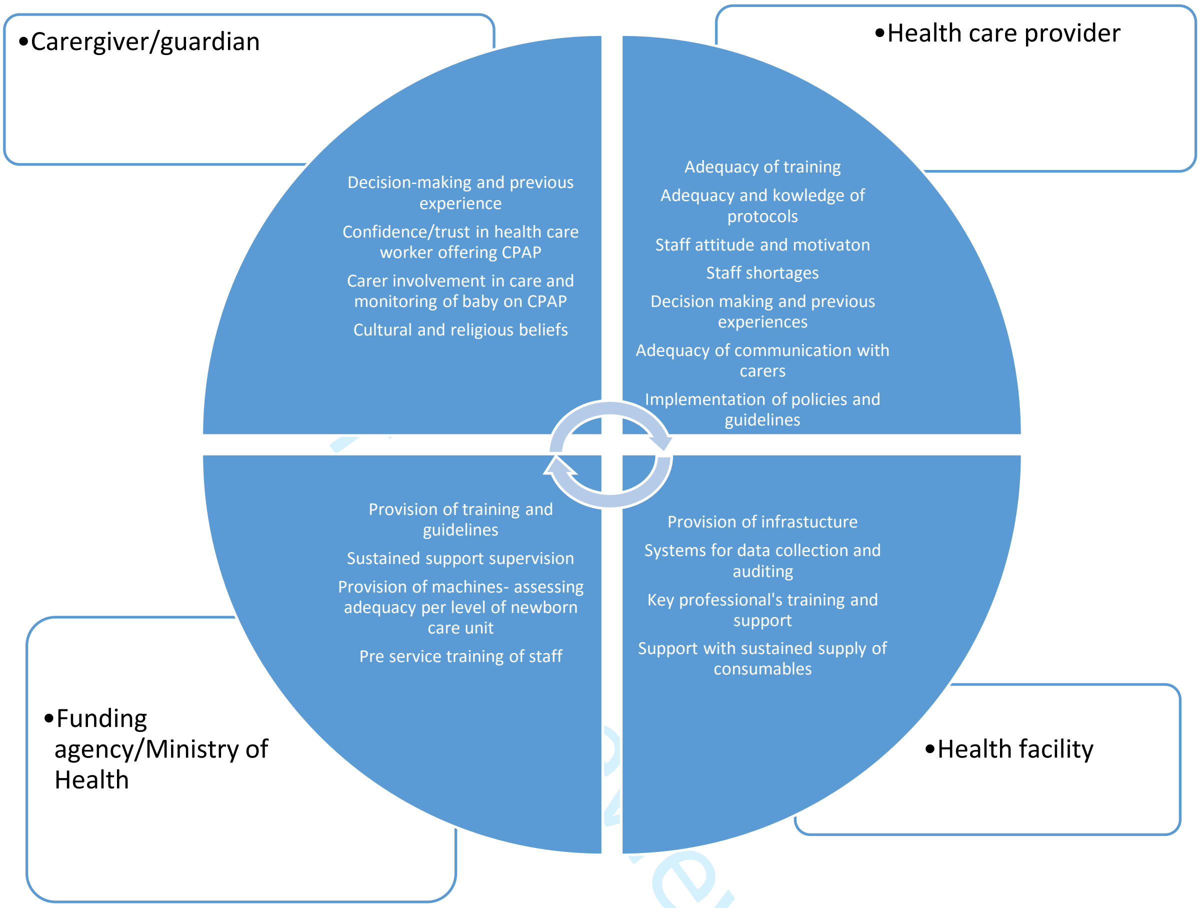

Reporting checklist for qualitative study.

Based on the SRQR guidelines.

Instructions to authors

Complete this checklist by entering the page numbers from your manuscript where readers will find each of the items listed below.

Your article may not currently address all the items on the checklist. Please modify your text to include the missing information. If you are certain that an item does not apply, please write "n/a" and provide a short explanation.

Upload your completed checklist as an extra file when you submit to a journal.

In your methods section, say that you used the SRQRreporting guidelines, and cite them as:

O'Brien BC, Harris IB, Beckman TJ, Reed DA, Cook DA. Standards for reporting qualitative research: a synthesis of recommendations. Acad Med. 2014;89(9):1245-1251.

		Reporting Item	Page Number
**Title**			
	#1	Concise description of the nature and topic of the study identifying the study as qualitative or indicating the approach (e.g. ethnography, grounded theory) or data collection methods (e.g. interview, focus group) is recommended	1
**Abstract**			
	#2	Summary of the key elements of the study using the abstract format of the intended publication; typically includes background, purpose, methods, results and conclusions	2-3
**Introduction**			
Problem formulation	#3	Description and ignificance of the problem / phenomenon studied: review of relevant theory and empirical work; problem statement	4-5

For peer review only

Purpose or research question	#4	Purpose of the study and specific objectives or questions	5
**Methods**			
Qualitative approach and research paradigm	#5	Qualitative approach (e.g. ethnography, grounded theory, case study, phenomenolgy, narrative research) and guiding theory if appropriate; identifying the research paradigm (e.g. postpositivist, constructivist / interpretivist) is also recommended; rationale. The rationale should briefly discuss the justification for choosing that theory, approach, method or technique rather than other options available; the assumptions and limitations implicit in those choices and how those choices influence study conclusions and transferability. As appropriate the rationale for several items might be discussed together.	6
Researcher characteristics and reflexivity	#6	Researchers' characteristics that may influence the research, including personal attributes, qualifications / experience, relationship with participants, assumptions and / or presuppositions; potential or actual interaction between researchers' characteristics and the research questions, approach, methods, results and / or transferability	8
Context	#7	Setting / site and salient contextual factors; rationale	6
Sampling strategy	#8	How and why research participants, documents, or events were selected; criteria for deciding when no further sampling was necessary (e.g. sampling saturation); rationale	7
Ethical issues pertaining to human subjects	#9	Documentation of approval by an appropriate ethics review board and participant consent, or explanation for lack thereof; other confidentiality and data security issues	5
Data collection methods	#10	Types of data collected; details of data collection procedures including (as appropriate) start and stop dates of data collection and analysis, iterative	8-9

For peer review only

		process, triangulation of sources / methods, and modification of procedures in response to evolving study findings; rationale	
Data collection instruments and technologies	#11	Description of instruments (e.g. interview guides, questionnaires) and devices (e.g. audio recorders) used for data collection; if / how the instruments(s) changed over the course of the study	8-9
Units of study	#12	Number and relevant characteristics of participants, documents, or events included in the study; level of participation (could be reported in results)	10
Data processing	#13	Methods for processing data prior to and during analysis, including transcription, data entry, data management and security, verification of data integrity, data coding, and anonymisation / deidentification of excerpts	8-9
Data analysis	#14	Process by which inferences, themes, etc. were identified and developed, including the researchers involved in data analysis; usually references a specific paradigm or approach; rationale	9
Techniques to enhance trustworthiness	#15	Techniques to enhance trustworthiness and credibility of data analysis (e.g. member checking, audit trail, triangulation); rationale	9
**Results/findings**			
Syntheses and interpretation	#16	Main findings (e.g. interpretations, inferences, and themes); might include development of a theory or model, or integration with prior research or theory	10-18
Links to empirical data	#17	Evidence (e.g. quotes, field notes, text excerpts, photographs) to substantiate analytic findings	11-18
**Discussion**			
Intergration with prior work, implications, transferability and contribution(s) to the field	#18	Short summary of main findings; explanation of how findings and conclusions connect to, support, elaborate on, or challenge conclusions of earlier scholarship; discussion of scope of application /	19-24

For peer review only

		generalizability; identification of unique contributions(s) to scholarship in a discipline or field	
Limitations	#19	Trustworthiness and limitations of findings	23-24
**Other**			
Conflicts of interest	#20	Potential sources of influence of perceived influence on study conduct and conclusions; how these were managed	23-24
Funding	#21	Sources of funding and other support; role of funders in data collection, interpretation and reporting	27

None The SRQR checklist is distributed with permission of Wolters Kluwer © 2014 by the Association of American Medical Colleges. This checklist can be completed online using https://www.goodreports.org/, a tool made by the EQUATOR Network in collaboration with Penelope.ai

For peer review only
